# Hydrothermally synthesized PZT film grown in highly concentrated KOH solution with large electromechanical coupling coefficient for resonator

**DOI:** 10.1098/rsos.171363

**Published:** 2017-12-20

**Authors:** Guo-Hua Feng, Kuan-Yi Lee

**Affiliations:** Department of Mechanical Engineering, National Chung Cheng University, Chiayi 621, Taiwan, Republic of China

**Keywords:** lead zirconate titanate, resonator, dome-shaped diaphragm, hydrothermal

## Abstract

This paper presents a study of lead zirconate titanate (PZT) films hydrothermally grown on a dome-shaped titanium diaphragm. Few articles in the literature address the implementation of hydrothermal PZT films on curved-diaphragm substrates for resonators. In this study, a 50-μm-thick titanium sheet is embossed using balls of designed dimensions to shape a dome-shaped cavity array. Through single-process hydrothermal synthesis, PZT films are grown on both sides of the processed titanium diaphragm with good adhesion and uniformity. The hydrothermal synthesis process involves a high concentration of potassium hydroxide solution and excess amounts of lead acetate and zirconium oxychloride octahydrate. Varied deposition times and temperatures of PZT films are investigated. The grown films are characterized by X-ray diffraction and scanning electron microscopy. The 10-μm-thick PZT dome-shaped resonators with 60- and 20-μm-thick supporting layers are implemented and further tested. Results for both resonators indicate that large electromechanical coupling coefficients and a series resonance of 95 MHz from 14 MHz can be attained. The device is connected to a complementary metal–oxide–semiconductor integrated circuit for analysis of oscillator applications. The oscillator reaches a *Q* value of 6300 in air. The resonator exhibits a better sensing stability when loaded with water when compared with air.

## 1. Introduction

Piezoelectric materials have been widely employed as actuators or sensors for exchange of electromechanical energy [1,2]. For example, quartz crystal resonators have been explored for several decades for online measurement of surface coverage by monolayers or for examining the energy-dissipating properties of bonded surface mass. The sensitivity of quartz crystal resonator sensors depends on fundamental frequency variations. The resonant frequency is associated with the thickness of the resonator. However, the challenge of fabricating thin-film quartz resonators still limits the improvement of the fundamental resonance.

Many research groups have reported that a piezoelectrically micromachined resonator exhibits remarkably high sensitivity because its volume and mass are lower than that of a quartz crystal resonator sensor by several orders of magnitude. Therefore, microelectromechanical system resonators have been thoroughly studied for applications such as gas identification and biological substance detection [3,4]. A popular type of microelectromechanical system piezoelectric resonator is made by using a piezoelectric film no thicker than a few micrometres, composed of substances such as lead zirconate titanate (PZT), aluminium nitride or zinc oxide, to form a film bulk acoustic wave resonator [5–8]. The resonator converts electrical energy into acoustic energy through an alternating electric field, and its resonant frequency depends on the thickness of the piezoelectric material. Moreover, these devices have the ability to generate a high resonant frequency and *Q* factor.

Although research on piezoelectrically micromachined devices is increasing, the applications of piezoelectric-film-based resonators as sensors are still being studied [9]. The energy dissipation from piezoelectric thin films was found to be comparable to air-damping, which lowers the *Q* factor of the device [10]. Furthermore, resonators with a thin planar film structure have a notably low resonant frequency and sensing ability, owing to a dramatic drop in the *Q* factor when the resonator is loaded with liquid samples [11]. Furthermore, many piezoelectric thin films on substrates are usually processed or annealed at high temperatures and consequently induce high residual stress [12,13]. This can deteriorate the piezoelectric driving capability of the fabricated device.

Therefore, this study deals with the aforementioned issues in the following ways: (1) relatively low processing temperature for fabrication of high-quality piezoelectric films on a micromachined resonator with minimized residual stress and uniform thickness formation and (2) increased stiffness of the resonator to overcome the damping effect caused by the film-based device interacting with air/liquid loading. Two schemes were employed in our investigation. The first one used a titanium dome-shaped diaphragm structure as the resonator substrate. The second one involved hydrothermal growth of PZT film on this titanium substrate; the entire fabrication process took place at temperatures lower than 200°C [14].

By forming piezoelectric films on dome-shaped diaphragm substrates, the produced residual stresses of piezoelectric films can be released through the curvature change of the dome-shaped diaphragm [13,15]. Meanwhile, a curved out-of-plane diaphragm possesses better stiffness than a flat diaphragm. According to the shadow shell theory, the natural frequency of a dome-like device can be derived as

1.1 $$F_{s}={\left[F_{f}^{2}+\frac{Y}{\rho {\left(2\pi \gamma \right)}^{2}}\right]}^{1/2},$$

where *F*_s_ and *F*_f_ represent the natural frequency of the shallow spherical shell and the flat plate, respectively. *Y* is Young's modulus, *ρ* is the material density and *γ* is the radius of curvature of the dome. This explains the extra rigidity of the out-of-plane structure; the increased stiffness results in a higher natural frequency of the dome-shaped structure. In addition, it is difficult to ensure uniform deposition of the PZT thin film on a dome-shaped diaphragm. The common sol–gel spray or spin-coating methods have limitations in the control of the thickness of deposition on the curved surfaces. It is also difficult to prepare high-quality polycrystalline PZT films by using sputtering or evaporation methods. Thus, we used hydrothermal synthesis for depositing the PZT films on the devices.

Literature on hydrothermal research can be dated back to the nineteenth century [16]. Hydrothermal synthesis has now become a promising technology for growing piezoelectric films. Some transducers with versatile piezoelectric films, composed of materials such as BaTiO_3_, PbTiO_3_, and PZT, have been fabricated using the hydrothermal method [17–20]. These hydrothermally realized transducers include an antiacoustic cavitation hydrophone and coiled-stator ultrasound micromotor [21,22]. The hydrothermal method has also been used for depositing crystalline PZT films with thicknesses of several micrometres on concave or convex Ti substrates at low temperatures [23]. The deposited PZT films exhibit good adhesion properties and require no further annealing for applications.

Several research groups have worked on the hydrothermal synthesis of PZT films. Abe *et al*. [16] presented hydrothermally synthesized PZT polycrystals deposited on a titanium substrate with source material ZrOCl_2_·8H_2_O/Pb(NO_3_)_2_/KOH/TiO_2_ = 60 ml (0.25 M)/100 ml (0.5 M)/200 ml (4 M)/1 g and synthesis conditions: 160°C, 0.5 MPa, stirring speed from 50 to 245 r.p.m., deposition time from 1 to 24 h. The results show the solution stirred at 245 r.p.m. and deposition for 5 h is an optimum condition. This causes approximately 20 µm thick PZT film with crystal size of 1.5 µm.

Suchanek & Riman [18] investigate the hydrothermal deposition of PZT films on single-crystal SrTiO_3_ (100) substrates. The reaction conditions of precursor concentration (Zr + Ti 0.3 M) and mineralizer concentration (KOH, 6 and 10 M) were chosen. All depositions were executed at a temperature of 150°C for 24 h. The stirring speed ranged from 0 to 1700 r.p.m. The results show that no film and a film thickness from 90 to 1225 nm were obtained. No film occurs at KOH of 6 M and stirring speed of 1700 r.p.m. The maximum thickness of 1225 nm occurs at KOH of 6 M and no stirring condition.

Ohba *et al*. [19] used two hydrothermal processes to fabricated PZT thin film on titanium film. The first process of PZT nucleation employed a solution of Pb(NO_3_)_2_, ZrOCl_2_ and KOH (8 M) with Pb/Zr molar ratio of 2.29. The deposition temperature and time are 150°C and 48 h, respectively. The second process of PZT crystal growth uses Pb(NO_3_)_2_, ZrOCl_2_, TiCl_4_ and KOH (4 M). The molar ratio of Pb : Zr : Ti is 110 : 52 : 48. The deposition temperature and time are 120°C and 24–120 h, respectively. The obtained PZT film thickness is from 10 to 23 µm with a gradually increasing trend when the deposition time of the second process is from 24 to 120 h. The density, Young's modulus and electromechanical coupling factor of PZT film are 4800 kg m^−3^, 42 GPa and 0.33, respectively.

You *et al*. hydrothermally grew PZT thin films on a titanium substrate at 160°C for 12 h. The precursor solution included ZrOCl_2_·8H_2_O (concentration 1.67 M)/TiCl_4_ (1.97 M)/Pb(NO_3_)_2_ (1 M)/KOH (8 M) [24]. The results show the PZT crystal displays a micrometre-sized hexahedron shape with well-developed facets. The chemical formula of the PZT crystal is expected to be PbZr_0.4_Ti_0.6_O_3_.

Compared to aforementioned works, our study uses relatively higher KOH concentration (10 M) and quite different ratio of Pb/Zr/Ti in precursor solution for hydrothermal synthesis of PZT film. The results show a 10 μm thick PZT film can be formed on the titanium sheet with pretty good polycrystalline structure when the deposition temperature is maintained at 180°C.

## 2. Device fabrication

### 2.1. Preparation of hydrothermal PZT film

We first prepared an aqueous solution of titanium dioxide. A 2.87 g mass of TiO_2_ powder was added to 350 g of deionized water and mixed by stirring for 10 min. After that, 27.07 g of zirconium oxychloride and 50.07 g of lead nitrate were added to the processed solution and stirred for 1 h. Then, 200 ml of 10 M potassium hydroxide solution was prepared and mixed with the solution containing TiO_2_/Zr. The molar ratio of Pb/Zr/Ti in the solution was 4.29 : 2.34 : 1. The solution colour became reddish brown after vigorous stirring for 1 h. The chemical reaction in this strongly alkaline solution could be described as follows:

2.1 $$\begin{array}{r} \text{KOH}\to {\text{K}}^{+}+\text{O}{\text{H}}^{-}, \end{array}$$

2.2 $$\begin{array}{r} \text{Pb}{\left(\text{N}{\text{O}}_{3}\right)}_{2}\to \text{P}{\text{b}}^{2+}+2\text{N}{{\text{O}}_{3}}^{-}, \end{array}$$

2.3 $$\begin{array}{r} \text{ZrOC}{\text{l}}_{2}\to \text{Zr}{\text{O}}^{2+}+2\text{C}{\text{l}}^{-}, \end{array}$$

2.4 $$\begin{array}{r} \text{Zr}{\text{O}}^{2+}+3\text{O}{\text{H}}^{-}+{\text{H}}^{+}\to \text{Zr}{\left(\text{OH}\right)}_{4}, \end{array}$$

2.5 $$\begin{array}{r} \text{Ti}{\text{O}}_{2}+2\text{O}{\text{H}}^{-}+2{\text{H}}^{+}\to \text{Ti}{\left(\text{OH}\right)}_{4} \end{array}$$

2.6 $$\begin{array}{r} \;\text{and}\;\text{P}{\text{b}}^{2+}+(1-x)\text{Z}{\text{r}}^{4+}+x\text{T}{\text{i}}^{4+}+6\text{O}{\text{H}}^{-}\to \text{Pb}(\text{Z}{\text{r}}_{1-x},\text{T}{\text{i}}_{x}){\text{O}}_{3}+3{\text{H}}_{2}\text{O}. \end{array}$$

**Figure 1.**
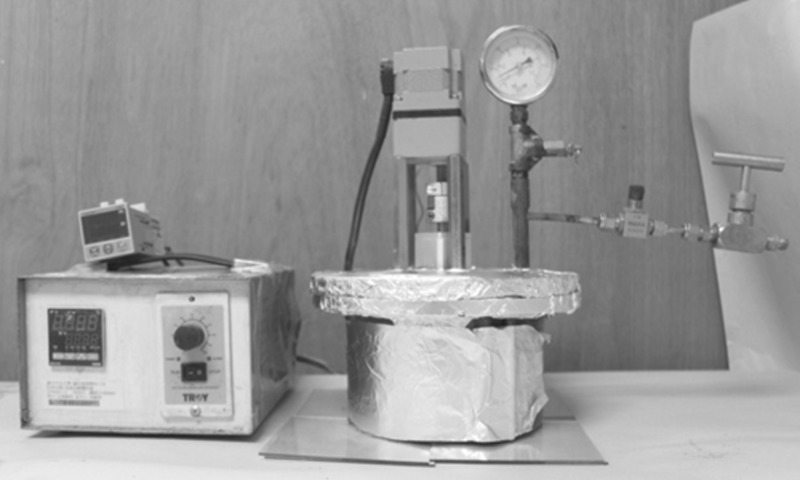
Experimental setup for hydrothermal growth of PZT film on titanium substrate.

A titanium substrate was then anchored on a metal holder and the prepared solution was poured into a home-made steel autoclave. The autoclave included a heating module, pressure gauge, temperature probe, pressure relief valve and rotation mechanism with a low-speed motor (figure 1). Apart from the heating module, which surrounded the lower chamber of the autoclave, the components were installed on the top cover of the autoclave. The holder with the titanium substrate was mounted on the rotation mechanism, such that the titanium substrate was fully immersed in the solution when the autoclave was sealed.

Different PZT deposition conditions using prepared solutions of the same compositions were studied. We heated the prepared solutions inside the autoclave individually from room temperature to temperatures of 160, 170, 180 and 190°C at a rate of 5°C min^−1^. The process temperatures were maintained at these values for 24, 36 and 48 h. Then, the heating function was turned off to allow the solution to cool to room temperature, and the titanium substrate with the grown PZT film was removed from the autoclave.

### 2.2. Fabrication of spherical-shaped piezoelectric resonators

**Figure 2.**
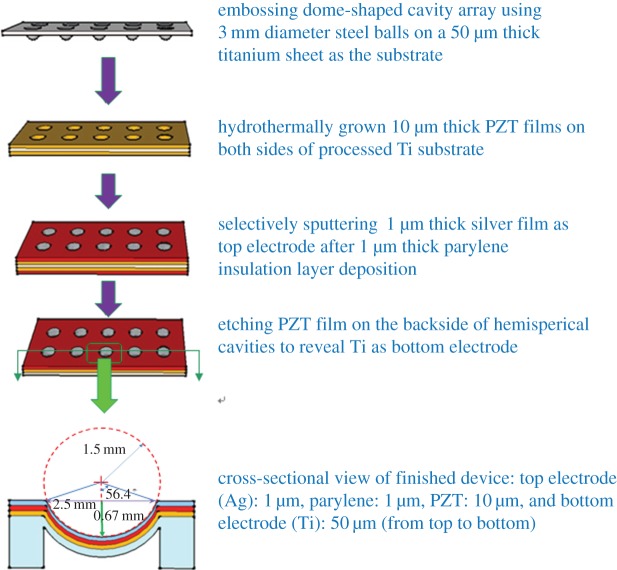
Fabrication process flow of dome-shaped titanium diaphragm resonator with hydrothermally grown PZT film.

To fabricate resonators using a thin titanium sheet, we first constructed dome-shaped structures on the titanium sheet by embossing (figure 2). The created structure was stiffer than the flat titanium sheet. We used steel balls 3 mm in diameter to press the titanium sheet in order to make an array of dome shape from the flat surface.

**Figure 3.**
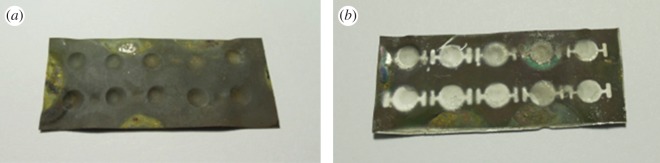
(*a*) Fabrication results of the hydrothermal PZT film deposited on the dome-shaped titanium diaphragm. (*b*) Top silver electrodes made on individual dome-shaped diaphragms by a shadow mask.

The processed titanium substrate was then subjected to hydrothermal PZT film growth with the aforementioned fabrication procedure; the results are shown in figure 3*a*. A shadow mask was designed and fabricated for selectively depositing the upper electrodes of the resonators. This was followed by sputtering 1-μm-thick silver film with a deposition power of 20 W for 15 min at an argon gas flow rate of 20 sccm (figure 3*b*). Since the titanium substrate served as the lower electrode in our resonator design to form a silver/PZT/titanium sandwich structure, we removed the lower side of the PZT film coated on the titanium substrate, so that a wire could be bonded to the lower electrode. This was handled by protecting the entire processed titanium substrate with photoresist, except for the lower part of the dome-shaped structure and the wire connection region. Then, a multi-metal etchant was applied to remove the unprotected PZT film with an etching rate of 1 µm min^−1^ at room temperature. After the PZT film was etched away, this etchant continued to etch the titanium supporting layer. Once the titanium thickness reached the required thickness, the protecting photoresist was stripped. Then, we bonded the wires to the electrodes, and the fabricated resonator was ready.

## 3. Microstructural characterization of hydrothermal PZT film

**Figure 4.**
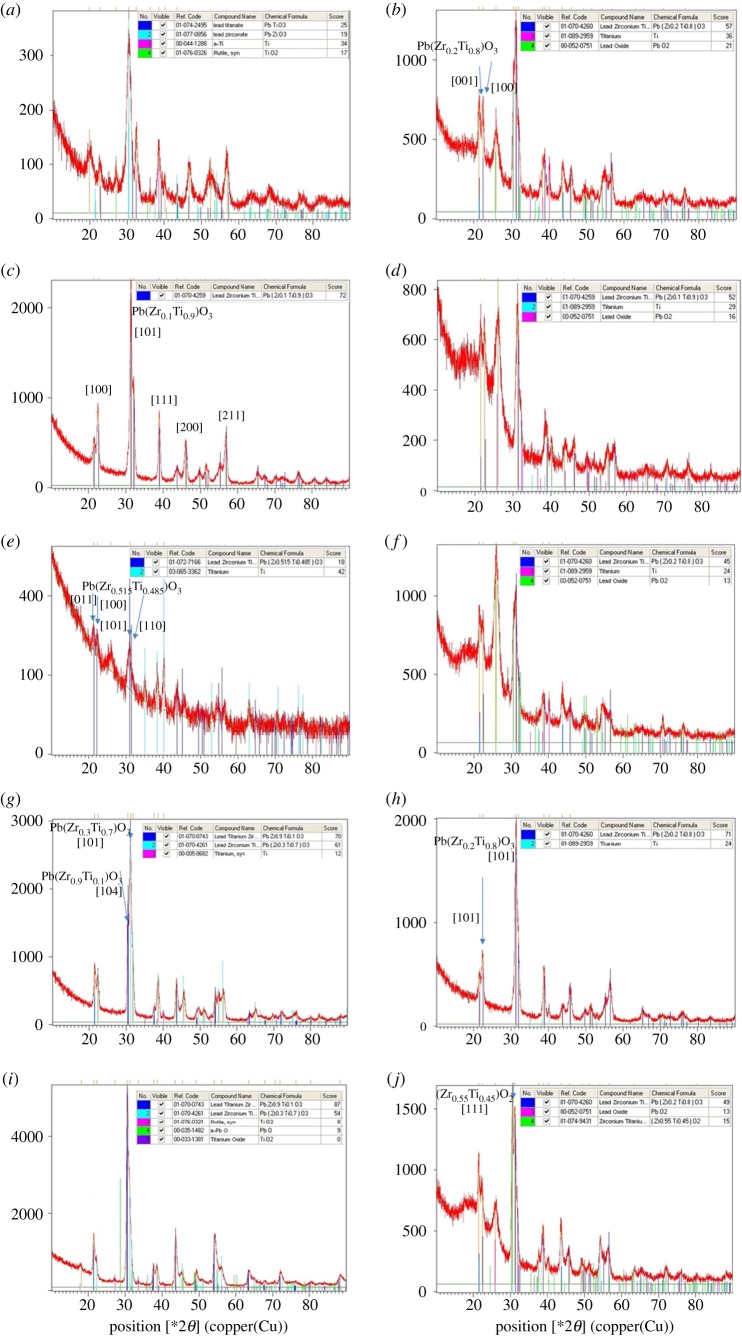
XRD patterns of hydrothermal PZT films for different temperature settings and different time periods of the deposition process. (*a*) 160°C, 24 h; (*b*) 170°C, 24 h; (*c*) 180°C, 24 h; (*d*) 190°C, 24 h; (*e*) 160°C, 36 h; (*f*) 170°C, 36 h; (*g*) 180°C, 36 h; (*h*) 190°C, 36 h; (*i*) 180°C, 48 h; (*j*) 190°C, 48 h.

Figure 4 shows the XRD patterns of hydrothermal PZT films for which the deposition process was maintained at different temperature settings and over different time periods. The characterization was performed on a PANalytical X'Pert Pro XRD system for a 2*θ* range from 5° to 90° with a scanning speed of 4° min^−1^ and an increment of 0.02°; CuK*α* radiation was employed (*λ *= 1.5406 nm).

PbO or PbO_2_ can be observed in the XRD results of the deposited films shown in figure 4*b*,*d*,*f*,*i* and *j*. This could be due to the use of an excessive amount of Pb, as measured by the molar ratio of the prepared solution. The form of lead oxide produced can vary depending on the feedstock pH value [24]. In the pH range of 8 to 10, the dominant species is PbOH^+^, which is replaced at a pH of approximately 11 by Pb(OH)_2_ (aq). Besides that, certain rutile TiO_2_ crystal peaks could be attributed to the presence of unreacted TiO_2_ compound in the solution.

Figure 4*a* indicates the presence of lead titanate and lead zirconate (of perovskite structure) with tetragonal and orthorhombic phases matching the JCPDS file nos. 74-2495 and 77-0856, respectively. According to the literature, the hydrothermal growth of PZT polycrystalline films on a titanium substrate involves nucleation and crystal growth. In the incipient stage of nucleation, the titanium substrate is attacked by the highly concentrated alkaline solution, and the surface titanium can be dissolved in the solution as titanium ions react with lead and zirconium ions. It seems that excess lead ions easily form PbTiO_3_ and PbZrO_3_ at low process temperatures.

The lead titanate and lead zirconate disappeared when the process temperature was raised to 170°C (figure 4*b*). However, the lead zirconate titanate perovskite structure of Pb(Zr_0.2_Ti_0.8_)O_3_ emerged with a tetragonal phase matching JCPDS no. 70-4260.

When the process temperature was increased to 180°C, sharp peaks of lead zirconium titanium oxide perovskite structure Pb(Zr_0.1_Ti_0.9_)O_3_ matching JCPDS no. 70-4256 were observed (figure 4*c*). The sharp peaks indicated the formation of larger grains with increased crystallinity. Further, impurities such as lead oxide disappeared. However, the even higher process temperature of 190°C did not contribute to sharper peaks and resulted in impurities in the deposited film such as PbO_2_ and Ti (figure 4*d*). The same composition of lead zirconium titanium oxide as that shown in figure 4*c* was observed.

When the film growth time was increased to 36 h (figure 4*e*–*h*), perovskite-structured PZT was observed for the four different process temperatures. The peak intensity was relatively small at 160°C compared to those at the other temperatures, probably because of the poor arrangement of atoms in the entire polycrystalline film (figure 4*e*). However, a Pb(Zr_0.515_Ti_0.485_)O_3_ polycrystalline film of tetragonal phase matching JCPDS no. 72-7166 was detected. This composition near the morphotropic phase boundary (MPB) possessed high piezoelectric response. In the neighbourhood of the MPB, the crystal structure changes rapidly and the electromechanical properties in piezoelectric materials are optimal [25,26].

For higher process temperatures, tetragonal Ti-rich PZT films were observed. Pb(Zr_0.2_Ti_0.8_)O_3_ polycrystalline film matching JCPDS no. 70-4260 was observed at 170°C and 190°C. Pb(Zr_0.3_Ti_0.7_)O_3_ film matching JCPDS no. 70-4261 was observed at 180°C. Sharp peaks with a relatively strong intensity were obtained at 180°C when compared with those at 170°C and 190°C.

When the deposition time was further increased to 48 h, the tetragonal Ti-rich PZT films Pb(Zr_0.3_Ti_0.7_)O_3_/ Pb(Zr_0.2_Ti_0.8_)O_3_ were still dominant. Meanwhile, Pb(Zr_0.9_Ti_0.1_)O_3_ polycrystalline film matching JCPDS no. 70-0743 with a sharp peak and high intensity was observed at 180°C; the peaks were sharper than those observed in the case of 36 h deposition time. At 190°C, Pb(Zr_0.2_Ti_0.8_)O_3_ film with the same composition as that observed with 36 h deposition was observed, but the emerging orthorhombic PbO_2_ and (Zr_0.55_Ti_0.45_)O_2_ structures downgraded the properties of the deposited PZT film.

**Figure 5.**
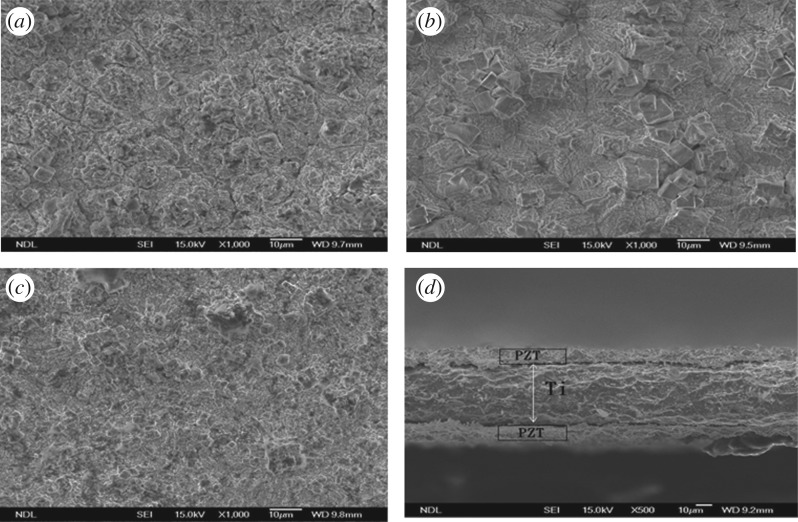
(*a*–*c*) SEM images of PZT films formed at 170°C, 180°C and 190°C at a deposition time of 24 h. (*d*) Cross-sectional view of the sample processed at 180°C for 48 h.

Figure 5*a*–*c* shows scanning electron microscope (SEM) images of PZT films formed at 170°C, 180°C, and 190°C for a deposition time of 24 h. The deposited films showed crystallization and had polycrystalline structure. In general, the grains were randomly oriented. Clear grain boundaries can be observed for the 170°C and 180°C samples. Some pores were found in the 170°C sample. For the 190°C sample, the grain size was relatively small, and the grain boundaries were not as obvious as in the other two cases. It is widely known that clear and uniform grain boundaries increase the mechanical strength of a film. Well-developed facets were easily observed in the 180°C sample, which exhibited high intensity and sharp peaks in the XRD results.

Based on the results shown in figure 5*a*–*c*, we found the sample processed at 180°C exhibited a better polycrystalline structure and grain boundaries compared to that processed at either lower temperature of 170°C or higher temperature of 190°C. Moreover, the sample processed with longer hours at a certain temperature condition exhibited a better polycrystalline structure. For example, the largest peak has the highest intensity compared to the samples deposited at 180°C for 24 h and 36 h.

Figure 5*d* shows the cross-sectional view of the sample processed at 180°C for 48 h. A 10-μm-thick PZT film was grown on both sides of a 50-μm-thick titanium sheet. The PZT film of the fabricated dome-shaped diaphragm resonator, which is discussed in the following sections, was based on this deposition condition.

## 4. Electrical impedance measurement and analysis

The fabricated resonator consisted of four layers: top silver electrode, parylene, PZT and bottom titanium electrode (figure 2). Unlike common thin-film based resonators, which have an individual bottom electrode and supporting layer/substrate, the resonator designed here had a titanium sheet substrate as the bottom electrode. The thick electrode layer provided independent support to the piezoelectric film, but the thickness was greater than that of a regular resonator. The parylene layer served as electrical insulation to increase the breakdown voltage when the fabricated device operated as a resonator. The resonator was subjected to measurements and analysis, as described in the following.

**Figure 6.**
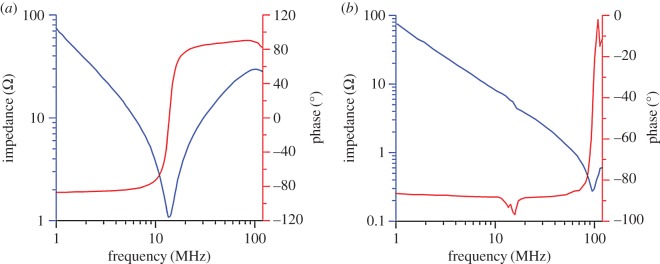
Results of measured electrical impedances of the fabricated dome-shaped diaphragm resonator with substrate thicknesses of (*a*) 60 µm and (*b*) 20 µm.

The impedance of the fabricated device was measured using an impedance analyzer (model 6500B, Wayne Kerr Electronics, UK). Figure 6*a* shows the device fabricated without any etching process to reduce the thickness of the backing PZT and titanium supporting layers, that is, the device was structured as PZT/Ti/PZT/parylene/Ag: 10/50/10/1/1 µm. Figure 6*b* shows the studied etching case with layers arranged with the Ti/PZT/parylene/Ag having thicknesses of 20 /10/1 /1 µm (after the backing PZT and partial titanium layers had been removed). The series (*f*_s_) and parallel (*f*_p_) resonant frequencies are 14.1 and 90.5 MHz, respectively, for the device without reducing the thickness of the backing PZT and Ti supporting layers. After the etching process to remove the backing PZT layer and reduce the Ti support layer to 20 µm, the series and parallel resonance frequencies are 95.2 and 116 MHz, respectively.

**Figure 7.**
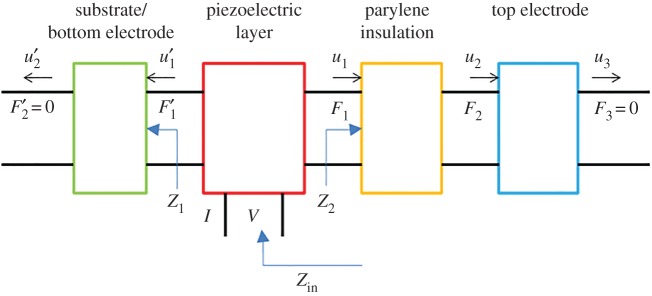
Schematic diagram of the analysed four-layer resonator using the Mason equivalent circuit model.

The etching process removes the backing PZT film and makes the titanium substrate become thinner, which results in higher series and parallel resonance frequencies. This can be evaluated by electrical impedance calculation. To simplify this analysis, we neglect the curvature effect and consider the planar device with the same layer composition as the fabricated device (figure 7). Also, the planar device operates in a thickness extension mode, such that the lateral dimension of the piezoelectric plate is greater than the thickness. Using the Mason model and transfer matrix method [27], we can derive the electrical impedance of the fabricated device looking into between the top and bottom surfaces of PZT film as

4.1 $$Z_{\mathrm{PZT}}^{e}=\frac{V}{I}=\frac{1}{j\omega C_{0}}\;\left[1-\frac{k_{t}^{2}}{\gamma }\frac{\left(z_{1}+z_{2})\;\sin\gamma +j2(1-\cos\gamma \right)}{(z_{1}+z_{2})\;\cos\gamma +j(1+z_{1}z_{2})\;\sin\gamma }\right],$$

where $z_{1}=Z_{1}/Z_{0}$ and $z_{2}=Z_{2}/Z_{0}$ are the normalized acoustic impedance on both sides of the PZT film. *Z*_2_ is the acoustic impedance of the parylene insulation and top electrode, *Z*_1_ the acoustic impedance of the bottom electrode, *Z*_0_ the acoustic impedance of the piezoelectric layer. γ=ωd/v is the phase delay of the acoustic wave in the PZT layer. *ω* is the angular velocity and *v* is propagation velocity. *d* is the thickness of PZT layer.

Moreover, the input impedance between two electrodes of the fabricated resonator can be calculated as

4.2 $$Z_{\mathrm{in}}^{e}=Z_{\mathrm{PZT}}^{e}+Z_{\mathrm{parylene}}^{e}=\frac{1}{j\omega }\;\left(\frac{1}{C_{0}}+\frac{1}{C_{\mathrm{parylene}}}\right)-\frac{1}{j\omega C_{0}}\;\left[\frac{k_{t}^{2}}{\gamma }\frac{\left(z_{1}+z_{2})\;\sin\gamma +j2(1-\cos\gamma \right)}{(z_{1}+z_{2})\;\cos\gamma +j(1+z_{1}z_{2})\;\sin\gamma }\right].$$

Using the parameters listed in table 1 for evaluation, the results indicate that the resonator structured with a 10-μm-thick PZT backside layer and a 50-μm-thick titanium layer has a series resonant frequency of 35.85 MHz and parallel resonant frequency of 35.91 MHz. If the backside PZT layer were totally etched away and the 20-μm-thick titanium supporting layer were left, the fundamental series and parallel resonant frequencies would become 78.72 and 79.75 MHz, respectively. It is obvious that the resonator with the reduced thickness of substrate possesses higher resonant frequency.

**Table 1.** Values used for calculation to derive the results shown in figure 6. Clamped capacitance of PZT film: 12.500 nF. Electromechanical coupling coefficient $k_{t}^{2}$ of PZT film: 0.793.

material layer	PZT	titanium	parylene	silver
acoustic impedance (MRayl)	32.6	27.3	2.6	38
acoustic velocity (m s^−1^)	4350	6100	2200	3600

**Figure 8.**
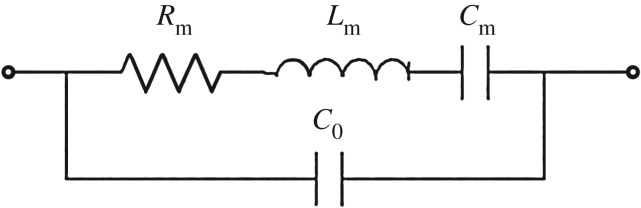
BVD equivalent circuit model to extract the parameters from the measured electrical impedance and electrical impedance calculated from the Mason circuit model.

We further employed a Butterworth–van Dyke (BVD) equivalent circuit model to analyse the difference in the electrical impedance for both cases. Figure 8 shows the BVD circuit with static capacitance *C*_0_, motional resistance *R*_m_, inductance *L*_m_ and capacitance *C*_m_. The static capacitance value *C*_0_ in the BVD circuit of the measurement case was considered to be the same value as that in the calculation case. Each corresponding element in the BVD circuit can be derived by the following equations through the series and parallel resonance frequencies obtained from the measurements:

4.3 $$f_{s}=\frac{1}{2\pi \sqrt{L_{m}C_{m}}}$$

and

4.4 $$f_{p}=\frac{1}{2\pi \sqrt{L_{m}(C_{0}C_{m}\text{/}(C_{0}+C_{m}))}}.$$

The resulting *R*_m_, *C*_m_ and *L*_m_ are listed in table 2. The calculated effective coupling factor $\left(K_{\mathrm{eff}}^{2}=(f_{p}^{2}-f_{s}^{2})/f_{p}^{2}\right)$ reaches 97.6% and 32.6% for the studied thick and thin substrate cases, respectively. The case of before etching exhibits larger motional resistance than the case of after etching. This could be induced by the internal friction and supporting loss. The ratio of the static capacitance to the motional capacitance is referred to as the *C* ratio (*C*_r_) and is a figure of merit of the resonator. A resonator with a small *C*_r_ is inductive for a large frequency band. This characteristic facilitates the design of wideband filters and allows oscillators to be pulled by external turning elements over the inductive frequency band [28].

**Table 2.** Key parameters derived from the BVD equivalent circuit.

parameter	backing PZT/Ti/PZT/parylene/Ag: 10 /50 /10 /1 /1 µm (before etching)	Ti/PZT/parylene/Ag: 20 /10/1/1 µm (after etching)
*R*_m_	1.01 Ω	0.28 Ω
*C*_m_	8164 pF	98.3 pF
*L*_m_	15.7 nH	28.5 nH
*f*_s_	14.1 MHz	95.2 MHz
*f*_p_	90.5 MHz	116 MHz
$K_{\mathrm{eff}}^{2}$	97.6%	32.6%
*Q*	1.38	60.9
*C*_r_	0.306	25.4

Since the motional capacitance represents the stiffness of the device, the resonator before etching can be inferred to have higher stiffness than that after etching. When the dome-shaped diaphragm device was loaded with a liquid, the high stiffness effectively prevented the rapid decay of the vibrational motion [29]. For instance, when using a resonator to detect bioparticles in a liquid sample, a strong damping effect due to the low stiffness of the resonator could significantly degrade the sensitivity.

Moreover, the piezoelectric coefficients of *d*_31_ and *d*_33_ of the fabricated PZT film could be estimated using the following equations [30,31]:

4.5 $$d_{33}=k_{33}{\left(\varepsilon_{33}^{T}\cdot s_{33}^{E}\right)}^{1/2}={\left[C^{T}\cdot \frac{t}{A_{e}}\cdot \frac{1}{4\rho \cdot f_{a}^{2}\cdot t^{2}}\cdot \frac{\pi \text{/}2\cdot f_{r}\text{/}f_{a}\cdot \tan(\pi \text{/}2\cdot (f_{a}-f_{r})\text{/}f_{a})}{1-\pi \text{/}2\cdot f_{r}\text{/}f_{a}\cdot \tan(\pi \text{/}2\cdot (f_{a}-f_{r})\text{/}f)}\right]}^{1/2}$$

and

4.6 $$d_{31}=k_{31}{\left(\varepsilon_{33}^{T}\cdot s_{11}^{E}\right)}^{1/2}={\left[C^{T}\cdot \frac{t}{A_{e}}\cdot \frac{1}{4\rho \cdot f_{r}^{2}\cdot L^{2}}\cdot \frac{\pi \text{/}2\cdot f_{a}\text{/}f_{r}}{\pi \text{/}2\cdot f_{a}\text{/}f_{r}-\text{tan}(\pi \text{/}2\cdot f_{a}\text{/}f_{r})}\right]}^{1/2},$$

where *k*_33_ and *k*_31_ are the electromechanical factors of the PZT film for longitudinal mode and transverse mode, respectively. $\varepsilon_{33}^{T}$ is the dielectric constant. $s_{33}^{E}$ and $s_{11}^{E}$ are the elastic compliances. *f*_r_ is the resonance frequency (for lossless case: *f*_r_ = *f*_s_). *f*_a_ is the antiresonance frequency (for lossless case: *f*_a_ = *f*_p_). *C*^T^ is the capacity measured at 1 kHz. *t* is the thickness of the PZT film. *A*_e_ is the effective area of the resonator and *L* is the characteristic length of the resonator. *ρ* is the density of the PZT material. Using the values t=10 μm, $C^{T}=10.87\;\text{nF}$, $\rho =6000\;\text{kg}\;{{\text{m}}^{-}}^{3}$, $A_{e}=8.85\;{\text{mm}}^{2}$, L=2.95 mm, $f_{r}=95.2\;\text{MHz}$, $f_{a}=116\;\text{MHz}$ to perform the calculation, we can obtain $d_{33}=470\;\text{pC}\;{\text{N}}^{-1}$ and $d_{31}=1.62\;\text{pC}\;{\text{N}}^{-1}$.

## 5. Performance of the resonator device as an oscillator

**Figure 9.**
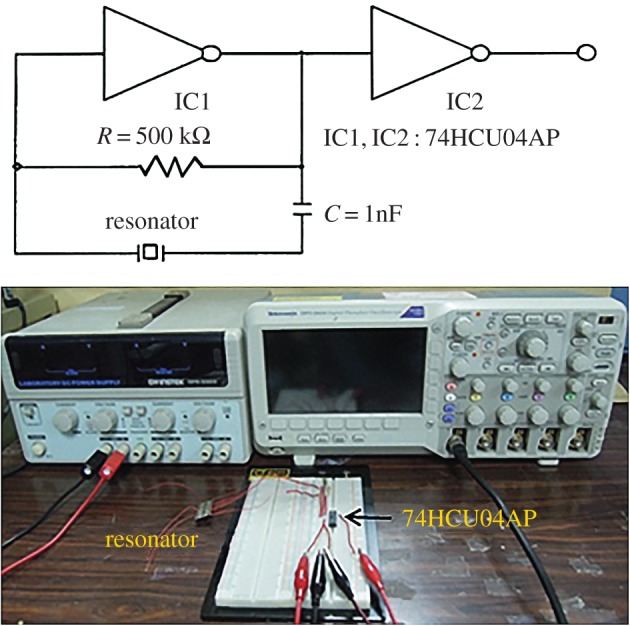
Experimental setup for characterizing the fabricated piezoelectric resonator as an oscillator.

To evaluate the performance of the fabricated resonator for oscillator applications, we constructed a circuit with two high-speed complementary metal–oxide–semiconductor invertors to form an oscillator for the tests (figure 9). We first recorded the output signal using a digital oscilloscope (Tektronix DPO2024, USA) at a sampling rate of 1 GHz, and employed a short time signal to find its frequency response. Then, the output signal was connected to a frequency counter (HP53131A Universal Frequency Counter) and transmitted to a computer through an RS232 cable to estimate the long-term characteristics.

Two experiments were then carried out. The first was to operate the device in air at room temperature. A thermocouple was placed in contact with the titanium substrate, which was located at the backside of device pad for wire connections. The detected temperature was recorded from the time the inverter power of the constructed circuit was turned on. The second experiment analysed the status of the cavity of the dome-shaped diaphragm transducer when it was filled with deionized water. This tested the transducer's efficacy as a sensor for detecting the properties or responses of a liquid.

**Figure 10.**
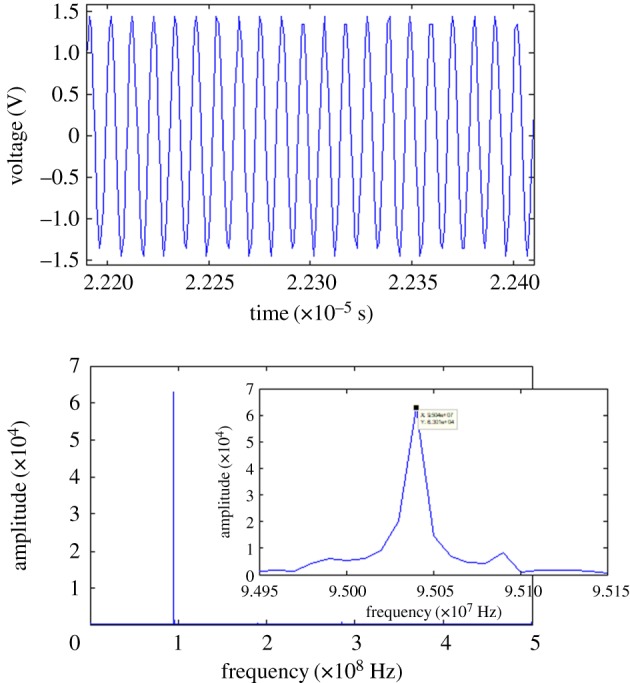
Results of the fabricated resonator frequency response based on a short-time analysis (0.1 ms) for testing in air.

Figure 10 shows the results of the frequency response of the fabricated resonator based on a short-time analysis (0.1 ms) for testing in air. The sinusoidal wave can be clearly observed. Through fast Fourier transform calculations, the resulting peak frequency was found to be 95.04 MHz and the −3 dB bandwidth was approximately 15 kHz. The corresponding quality factor was 6336.

According to literature data, a common commercial quartz resonator operating in air at 10 MHz has a quality factor of 6533 (approximately 7000) [32,33]. The quality factors of the micromachined 10 µm thick quartz resonator operated at 100 MHz display a value of 780 for the resonator without peripheral electrode and a value of 17 900 for the resonator with electrode optimization [34]. Also, the 52.6 MHz flexure-based mode of the 0.5 µm PZT on 4 µm silicon resonator is reported to have a quality factor of 122 [35]. Compared to aforementioned works, our resonator with *Q* value of 6336 is similar to the commercial quartz resonator operated at 10 MHz. In terms of operation at approximately 100 MHz, our *Q* value is better than the *Q* value of the micromachined quartz resonator without peripheral electrode but lower than that with electrode optimization. Regarding the PZT film constructed resonators operated at tens of MHz, the quality factor of our resonator exhibits a superior performance compared to that of the flexure-based mode of the silicon resonator.

**Figure 11.**
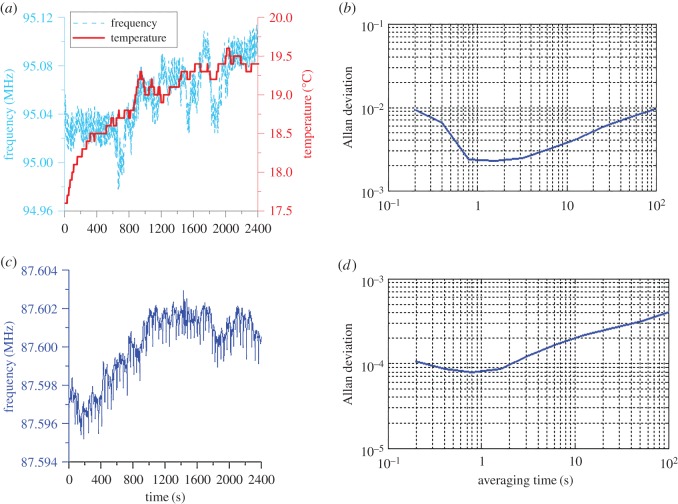
Long-term operation (40 min) results: (*a*) testing case in air; (*b*) Allan deviation of the measured results shown in (*a*); (*c*) testing results when filled with deionized water at room temperature; (*d*) Allan deviation of the measured results shown in (*c*).

Figure 11 shows the results for long-term operation (40 min). Figure 11*a* shows that the gauged temperature started from 17.5°C to 19.5°C and reached a nearly steady state at approximately 30 min for testing in air. The temperature rise could be attributed to the heating effect of the electrical junction resistance between the device connection pad and the wire during resonator operation. The sensed resonant frequency ranged from 94.98 to 95.11 MHz. This variation could be attributed to the slightly increased temperature and uncontrolled surrounding air flow.

The Allan deviation is used for measuring the frequency stability in an oscillator by analysing a time-domain data sequence. This scheme can be used to determine the intrinsic noise in the oscillating system as a function of the average time. Figure 11*b* shows the Allan deviation of the measured results shown in figure 11*a*.

The minimum value of the Allan deviation (2.2 × 10^−3^) was obtained for an observation time of 1 s. This indicates that the instability in frequency between two observations one second apart had a relative root mean square value of 2.2 kHz.

Figure 11*c* shows the testing results when the device was filled with deionized water at room temperature. The detected frequency ranged from 87.595 to 87.603 MHz for 40 min measurement, which exhibited a lower variation than the experiment in air. Based on the Allan deviation analysis, the minimum value was 8 × 10^−5^ at approximately 1 s (figure 11*d*). This indicated an optimal frequency instability of 80 Hz within 1 s.

## 6. Conclusion

Using the precursor solution of an excess amount of lead acetate and zirconium oxychloride octahydrate along with the high concentration of KOH solution, the hydrothermal growth of PZT films on dome-shaped titanium diaphragms is investigated. The resulting PZT films show a ferroelectric tetragonal phase at process temperatures between 170°C and 190°C. Besides that, the sample processed at 180°C exhibited a better polycrystalline structure and grain boundaries. The formed PZT film is prone to a Ti-rich phase and exhibits a preferred [101] orientation in our studied single-deposition process.

The fabricated resonators demonstrate that an increase of 81 MHz can be attained from a series resonance of 14 MHz after removing the backing PZT film and reducing Ti substrate to 20 µm. The estimated piezoelectric constants *d*_33_ and *d*_31_ of the fabricated PZT film are 470 and 1.62 pC N^−1^, respectively. According to the extracted parameters of the equivalent BVD circuit model, the coupling factors ($K_{\mathrm{eff}}^{2}$) reach 97.6% and 32.6% with corresponding *Q* values of 1.38 and 60.9 for the two studied cases, respectively. When the resonator was used as an oscillator, the minimum Allan deviation values were 2.2 kHz and 80 Hz for 40 min tests in air and water, respectively.

## Supplementary Material

XRD code dataset

## Supplementary Material

XRD code dataset

## Supplementary Material

XRD code dataset

## Supplementary Material

XRD code dataset

## Supplementary Material

XRD code dataset

## Supplementary Material

XRD code dataset

## Supplementary Material

XRD code dataset

## Supplementary Material

XRD code dataset

## Supplementary Material

XRD code dataset

## Supplementary Material

XRD code dataset

## Supplementary Material

XRD code dataset

## Supplementary Material

XRD code dataset

## Supplementary Material

XRD code dataset

## Supplementary Material

XRD code dataset

## Supplementary Material

XRD code dataset

## Supplementary Material

XRD code dataset

## Data accessibility

The datasets supporting this article have been uploaded as part of the electronic supplementary material.
