# Supplementary material for: Hydrothermally synthesized PZT film grown in highly concentrated KOH solution with large electromechanical coupling coefficient for resonator
Source: R Soc Open Sci. 2017 Dec 20;4(12):171363. doi: 10.1098/rsos.171363 (PMC5750027; doi:10.1098/rsos.171363)

Name and formula

Reference code:	01-074-9431
Compound name:	Zirconium Titanium Oxide
Empirical formula:	$O_2Ti_{0.45}Zr_{0.55}$
Chemical formula:	$(Zr_{0.55}Ti_{0.45})O_2$

Crystallographic parameters

Crystal system:	Orthorhombic
Space group:	Pbcn
Space group number:	60
a (A):	4.8349
b (A):	5.4801
c (A):	5.0439
Alpha (°):	90.0000
Beta (°):	90.0000
Gamma (°):	90.0000
Volume of cell (10^6 pm^3):	133.64
Z:	4.00
RIR:	6.45

Subfiles and quality

Subfiles:	ICSD Pattern
	Inorganic
Quality:	Star (S)

Comments

ANX:	AX2
ICSD collection code:	153940
Creation Date:	7/26/2010
Modification Date:	1/17/2013
ANX:	AX2
Analysis:	O2 Ti0.45 Zr0.55
Formula from original source:	(Zr0.55 Ti0.45) O2
ICSD Collection Code:	153940
Wyckoff Sequence:	d c(PBCN)
Unit Cell Data Source:	Powder Diffraction.

References

Primary reference: *Calculated from ICSD using POWD-12++*
Structure: Troitzsch, U., Christy, A.G., Ellis, D.J., *Phys. Chem. Miner.*, **32**, 504, (2005)

Peak list

No.	h	k	l	d [A]	2Theta[deg]	I [%]
1	1	1	0	3.62560	24.533	12.4
2	1	1	1	2.94390	30.337	100.0
3	0	2	0	2.74000	32.655	10.2
4	0	0	2	2.52200	35.568	8.4
5	2	0	0	2.41740	37.162	4.9
6	0	2	1	2.40770	37.318	5.1
7	1	0	2	2.23600	40.302	2.7
8	1	2	1	2.15530	41.881	6.5
9	1	1	2	2.07030	43.687	2.8
10	2	1	1	2.02560	44.702	0.1
11	0	2	2	1.85560	49.054	10.2
12	2	2	0	1.81280	50.292	10.2
13	2	0	2	1.74520	52.384	15.9
14	1	2	2	1.73240	52.801	0.3
15	1	3	0	1.70880	53.588	7.2
16	2	2	1	1.70590	53.686	10.3
17	2	1	2	1.66290	55.191	0.1
18	1	3	1	1.61840	56.844	5.7
19	3	1	0	1.54620	59.761	0.2
20	1	1	3	1.52530	60.665	11.0
21	3	1	1	1.47830	62.808	9.9
22	2	2	2	1.47200	63.108	5.6
23	0	2	3	1.43300	65.033	3.7
24	1	3	2	1.41460	65.986	5.2
25	2	3	1	1.40010	66.758	0.1
26	1	2	3	1.37400	68.198	0.1
27	0	4	0	1.37000	68.425	0.1
28	3	0	2	1.35800	69.115	0.2
29	2	1	3	1.33930	70.220	0.6
30	3	2	1	1.33930	70.220	0.6
31	0	4	1	1.32210	71.272	3.3
32	3	1	2	1.31820	71.515	0.9
33	1	4	1	1.27530	74.316	0.2
34	2	3	2	1.26100	75.304	1.2
35	0	0	4	1.26100	75.304	1.2
36	2	2	3	1.23270	77.348	0.3
37	1	0	4	1.22020	78.291	0.3
38	3	2	2	1.21680	78.552	0.1
39	4	0	0	1.20870	79.181	3.0
40	3	3	0	1.20870	79.181	3.0
41	0	4	2	1.20390	79.559	0.6
42	1	3	3	1.19850	79.990	1.3
43	1	1	4	1.19190	80.523	0.9
44	2	4	0	1.19190	80.523	0.9
45	3	3	1	1.17520	81.910	1.2
46	1	4	2	1.16820	82.507	0.2
47	2	4	1	1.16000	83.219	2.7
48	4	1	1	1.14930	84.170	0.1
49	0	2	4	1.14550	84.514	1.2
50	3	1	3	1.13810	85.193	2.6
51	2	0	4	1.11800	87.102	1.6
52	1	2	4	1.11460	87.434	0.1

53	4	2	0	1.10590	88.300	0.8
54	2	1	4	1.09540	89.371	0.1
55	3	3	2	1.08980	89.955	2.7
56	4	0	2	1.08980	89.955	2.7
57	4	2	1	1.08020	90.977	0.6
58	2	4	2	1.07760	91.258	0.1
59	4	1	2	1.06890	92.216	1.2
60	1	5	0	1.06890	92.216	1.2
61	0	4	3	1.06210	92.981	0.7
62	1	5	1	1.04570	94.891	0.1
63	2	2	4	1.03520	96.165	1.5
64	3	4	1	1.02220	97.801	0.1
65	1	3	4	1.01460	98.790	1.4
66	4	2	2	1.01280	99.028	1.7
67	3	0	4	0.99310	101.728	0.1
68	4	3	1	0.98850	102.386	0.1
69	1	5	2	0.98420	103.011	2.1
70	3	3	3	0.98130	103.437	1.0
71	3	1	4	0.97720	104.049	0.2
72	2	4	3	0.97240	104.776	3.0
73	1	1	5	0.97240	104.776	3.0
74	4	1	3	0.96610	105.752	0.1
75	3	4	2	0.96450	106.003	0.1
76	5	1	0	0.95230	107.974	0.2
77	0	2	5	0.94670	108.912	0.1
78	4	3	2	0.93570	110.820	1.4
79	5	1	1	0.93570	110.820	1.4
80	1	2	5	0.92900	112.027	0.1
81	0	4	4	0.92780	112.248	0.1
82	2	5	2	0.92780	112.248	0.1
83	4	2	3	0.92390	112.971	1.2
84	2	1	5	0.91780	114.130	0.1
85	1	4	4	0.91120	115.423	0.1
86	3	5	0	0.90630	116.410	1.0
87	4	4	0	0.90630	116.410	1.0
88	5	0	2	0.90290	117.110	0.1
89	0	6	1	0.89870	117.991	0.9
90	5	2	1	0.89730	118.289	0.1
91	3	5	1	0.89210	119.417	1.6
92	4	4	1	0.89210	119.417	1.6
93	3	4	3	0.88680	120.599	0.1
94	1	6	1	0.88360	121.331	0.1
95	2	2	5	0.88150	121.819	1.1
96	3	3	4	0.87250	123.980	1.7
97	4	0	4	0.87250	123.980	1.7
98	1	3	5	0.86870	124.930	0.6
99	2	4	4	0.86620	125.567	0.2
100	4	1	4	0.86170	126.743	0.1
101	2	5	3	0.85880	127.520	0.2
102	0	6	2	0.85880	127.520	0.2
103	5	2	2	0.85750	127.873	0.1
104	5	3	0	0.85460	128.674	0.7
105	2	6	0	0.85460	128.674	0.7
106	3	5	2	0.85290	129.152	1.6
107	4	4	2	0.85290	129.152	1.6
108	3	1	5	0.84490	131.484	1.5
109	2	6	1	0.84240	132.244	1.6
110	5	3	1	0.84240	132.244	1.6
111	0	0	6	0.84060	132.802	0.6
112	4	2	4	0.83140	135.794	1.0

113	1	0	6	0.82860	136.758	1.6
114	5	1	3	0.82860	136.758	1.6
115	1	1	6	0.81890	140.323	0.2
116	1	5	4	0.81540	141.709	1.4
117	0	4	5	0.81230	142.990	0.9
118	5	3	2	0.80940	144.237	0.8
119	2	6	2	0.80940	144.237	0.8
120	6	0	0	0.80580	145.859	0.2
121	0	2	6	0.80370	146.848	0.4
122	3	4	4	0.80370	146.848	0.4
123	0	6	3	0.80260	147.380	0.5
124	3	5	3	0.79780	149.825	0.9
125	4	4	3	0.79780	149.825	0.9

Stick Pattern

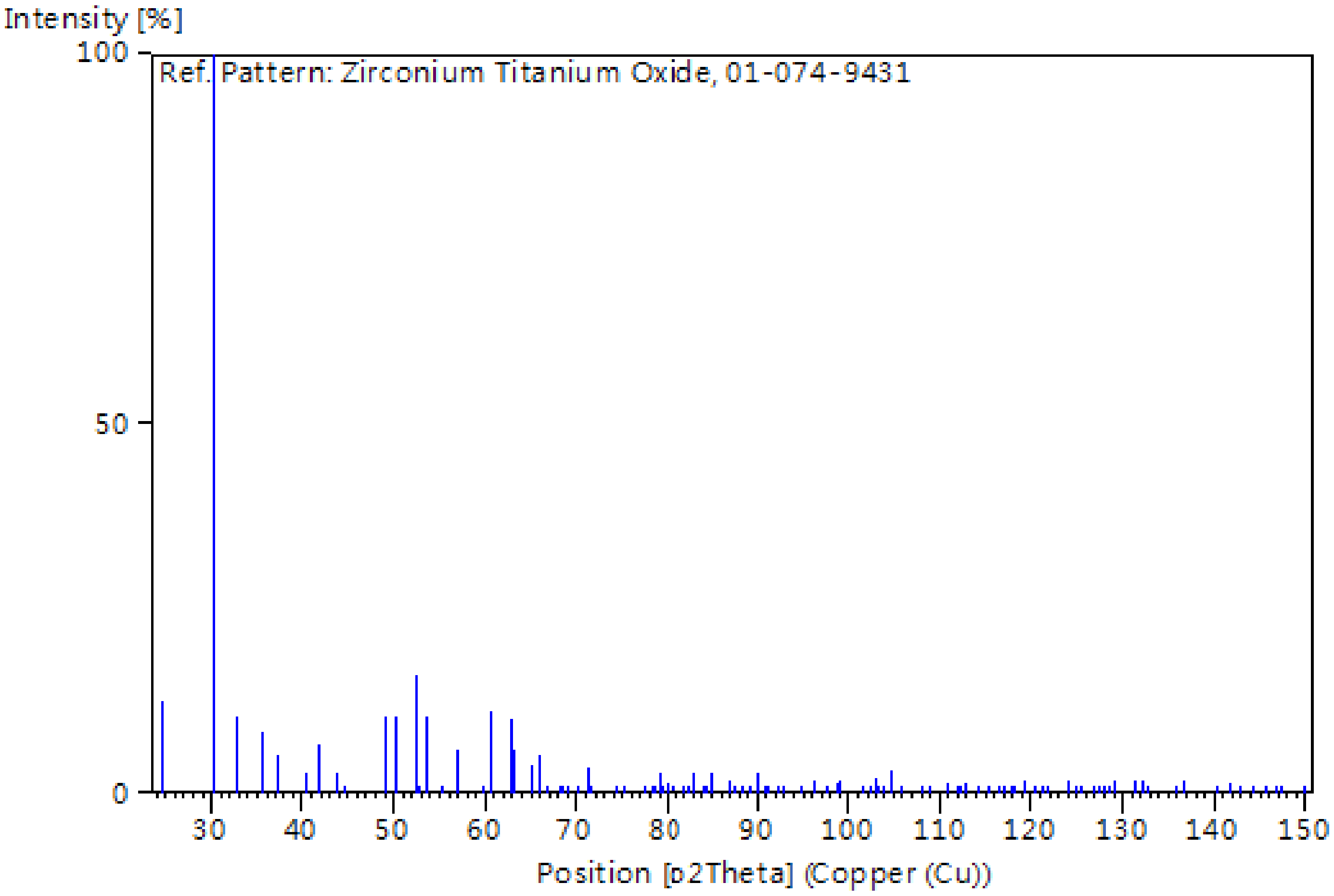

Supplement: XRD code dataset [file rsos171363supp1.pdf]
