# Supplementary material for: Hydrothermally synthesized PZT film grown in highly concentrated KOH solution with large electromechanical coupling coefficient for resonator
Source: R Soc Open Sci. 2017 Dec 20;4(12):171363. doi: 10.1098/rsos.171363 (PMC5750027; doi:10.1098/rsos.171363)

Name and formula

Reference code:	00-052-0751
Compound name:	Lead Oxide
Empirical formula:	O_2Pb
Chemical formula:	PbO_2

Crystallographic parameters

Crystal system:	Orthorhombic
Space group:	Pnnm
Space group number:	58
a (A):	4.9300
b (A):	4.8140
c (A):	3.3630
Alpha (°):	90.0000
Beta (°):	90.0000
Gamma (°):	90.0000
Volume of cell (10^6 pm^3):	79.81
Z:	2.00
RIR:	-

Status, subfiles and quality

Status:	Diffraction data collected at non ambient pressure
Subfiles:	Alloy, metal or intermetalic
	Inorganic
Quality:	Star (S)

Comments

Color:	Red
Creation Date:	2/2/1998
Modification Date:	1/12/2013
Color:	Red
General Comments:	Phase I`
Sample Preparation:	Forms from rutile type "O2 Pb" above 7.3 GPa and 200 C
Sample Source or Locality:	Commercial sample from Alfa Products
Unit Cell Data Source:	Powder Diffraction.

References

Primary reference: Haines, J., Leger, J., Schulte, O., *J. Phys.: Condens. Matter*, **8**, 1631, (1996)

Peak list

No.	h	k	l	d [A]	2Theta[deg]	I [%]
1	1	1	0	3.44400	25.849	100.0
2	1	0	1	2.77800	32.197	43.0
3	0	1	1	2.75700	32.449	43.0
4	2	0	0	2.46500	36.419	20.0
5	0	2	0	2.40700	37.329	9.0
6	2	1	1	1.83800	49.555	29.0
7	1	2	1	1.81900	50.108	29.0
8	2	2	0	1.72200	53.145	14.0
9	0	0	2	1.68200	54.512	6.0
10	3	1	0	1.55500	59.388	11.0
11	1	3	0	1.52600	60.634	9.0
12	1	1	2	1.51100	61.300	17.0
13	3	0	1	1.47600	62.917	12.0
14	0	3	1	1.44800	64.278	6.0
15	2	0	2	1.38900	67.362	5.0
16	0	2	2	1.37900	67.917	4.0
17	3	2	1	1.25900	75.445	7.0
18	2	3	1	1.24900	76.156	6.0
19	2	2	2	1.20300	79.631	8.0
20	3	3	0	1.14800	84.288	3.0
21	3	1	2	1.14200	84.834	6.0
22	1	3	2	1.13000	85.950	6.0
23	4	1	1	1.12500	86.426	6.0
24	1	4	1	1.10400	88.491	5.0
25	4	2	0	1.09700	89.206	3.0

Stick Pattern

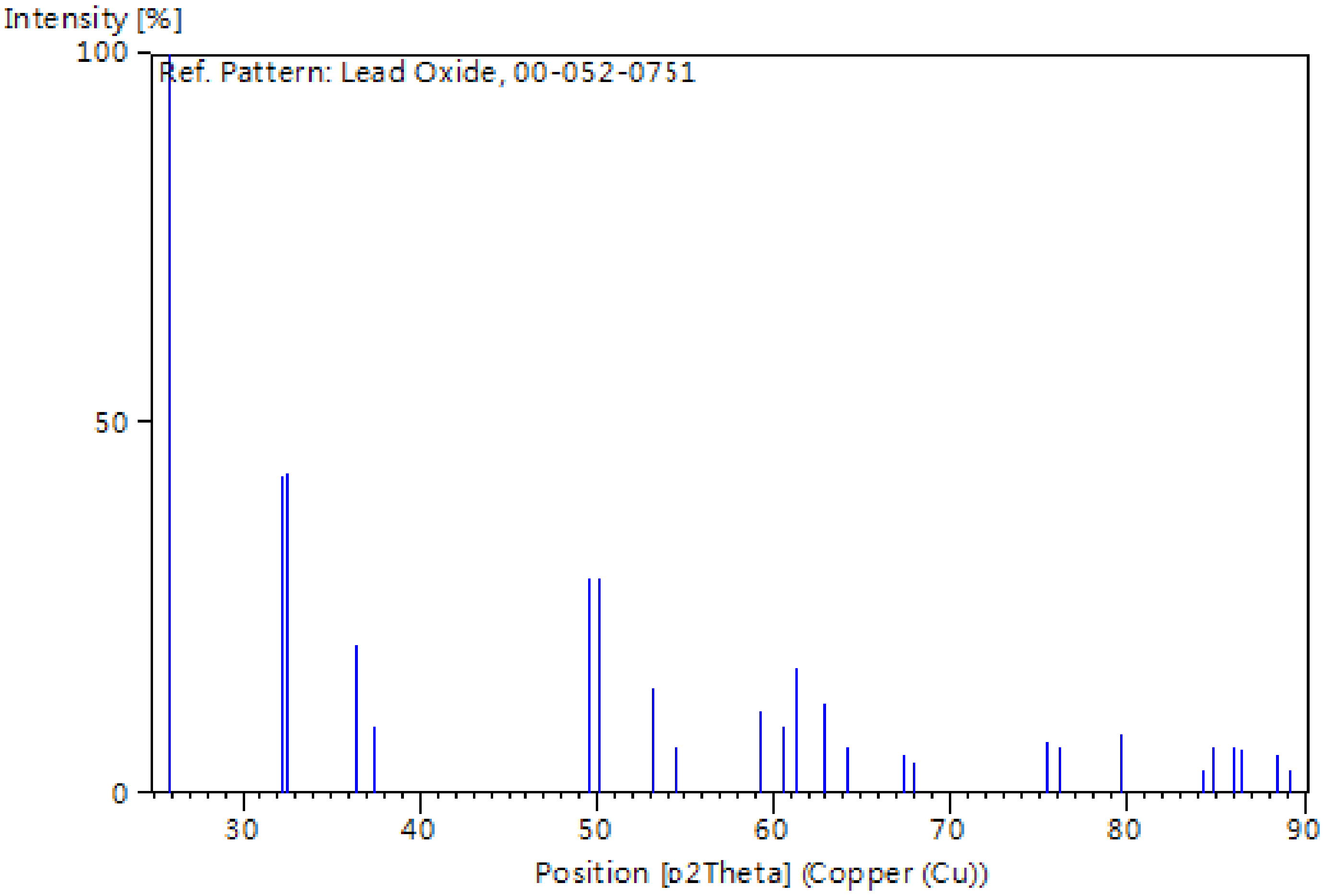

Supplement: XRD code dataset [file rsos171363supp2.pdf]
