# Supplementary material for: Hydrothermally synthesized PZT film grown in highly concentrated KOH solution with large electromechanical coupling coefficient for resonator
Source: R Soc Open Sci. 2017 Dec 20;4(12):171363. doi: 10.1098/rsos.171363 (PMC5750027; doi:10.1098/rsos.171363)
Supplement: XRD code dataset [file rsos171363supp3.pdf]

Name and formula

Reference code:	00-035-1482
Compound name:	Lead Oxide
Common name:	a-Pb O
Empirical formula:	OPb
Chemical formula:	PbO

Crystallographic parameters

Crystal system:	Orthorhombic
Space group:	Cmma
Space group number:	67
a (A):	5.6085
b (A):	5.6036
c (A):	4.9893
Alpha (°):	90.0000
Beta (°):	90.0000
Gamma (°):	90.0000
Volume of cell (10^6 pm^3):	156.80
Z:	4.00
RIR:	-

Subfiles and quality

Subfiles:	Alloy, metal or intermetalic
	Common Phase
	Educational pattern
	Inorganic
	Superconducting Material
Quality:	Star (S)

Comments

Color:	Violet-red
Creation Date:	12/14/1984
Modification Date:	1/11/2013
Additional Patterns:	See PDF 01-078-1663, 01-078-1664, 01-078-1665 and 01-078-1666
Color:	Violet-red
Note:	Low temperature phase-transition
Sample Preparation:	Preparation by thermal decomposition of lead dioxide a-"Pb O2" (Merck) at 793 K
Unit Cell:	a-PbO = a-PbO orthorhombic at 200 K.

References

Primary reference: Boher, P., *Private Communication*, (1984)
Unit cell: Boher, P., Garnier., *C. R. Seances Acad. Sci., Ser. 2*, **298**, 203, (1984)

Peak list

No.	h	k	l	d [A]	2Theta[deg]	I [%]
1	0	0	1	4.98930	17.763	3.0
2	1	1	1	3.10370	28.741	100.0
3	2	0	0	2.80420	31.888	16.0
4	0	2	0	2.80180	31.916	16.0
5	0	0	2	2.49460	35.972	11.0
6	2	0	1	2.44460	36.734	1.0
7	0	2	1	2.44300	36.759	1.0
8	1	1	2	2.11130	42.796	1.0
9	2	2	0	1.98200	45.741	16.0
10	2	0	2	1.86390	48.821	13.0
11	0	2	2	1.86320	48.841	13.0
12	2	2	1	1.84200	49.440	1.0
13	3	1	1	1.67100	54.901	16.0
14	1	3	1	1.66990	54.940	16.0
15	0	0	3	1.66310	55.184	1.0
16	2	2	2	1.55180	59.523	11.0
17	1	1	3	1.53360	60.302	11.0
18	3	1	2	1.44540	64.408	1.0
19	1	3	2	1.44470	64.443	1.0
20	2	0	3	1.43040	65.166	1.0
21	0	2	3	1.43010	65.181	1.0
22	4	0	0	1.40210	66.650	2.0
23	0	4	0	1.40090	66.715	2.0
24	4	0	1	1.34980	69.595	1.0
25	0	4	1	1.34870	69.660	1.0
26	3	3	1	1.27730	74.180	6.0
27	2	2	3	1.27400	74.405	1.0
28	4	2	0	1.25390	75.806	3.0
29	2	4	0	1.25320	75.855	3.0
30	0	0	4	1.24730	76.278	1.0
31	4	0	2	1.22230	78.131	2.0
32	0	4	2	1.22150	78.191	2.0
33	4	2	1	1.21600	78.613	1.0
34	2	4	1	1.21550	78.652	1.0
35	3	1	3	1.21310	78.838	5.0
36	1	3	3	1.21270	78.869	5.0
37	1	1	4	1.18980	80.695	1.0
38	3	3	2	1.16770	82.550	1.0
39	2	0	4	1.13970	85.045	2.0
40	0	2	4	1.13950	85.063	2.0
41	4	2	2	1.12030	86.878	4.0
42	2	4	2	1.11980	86.927	4.0

Stick Pattern

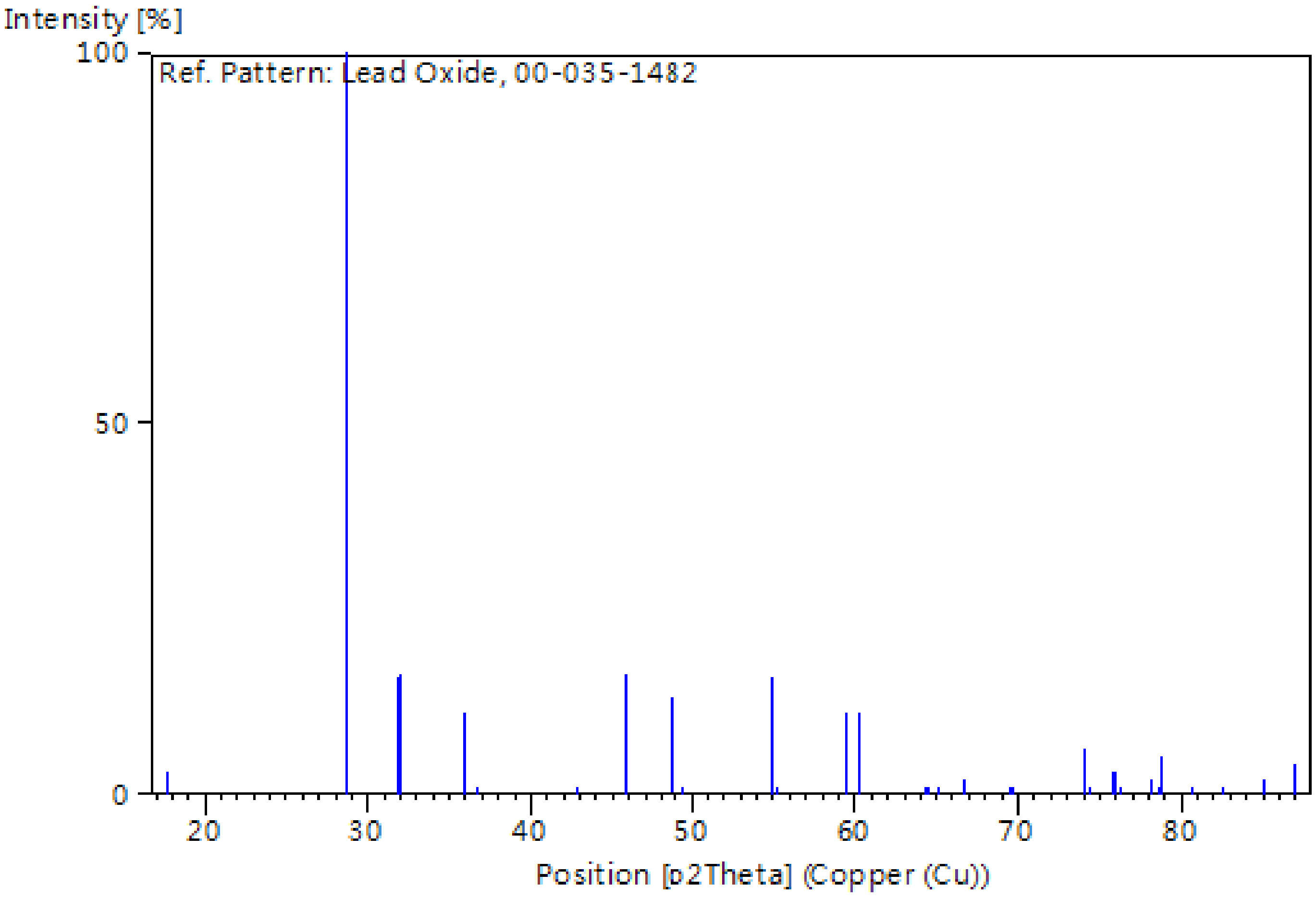
Intensity [%]
100
50
0
Ref. Pattern: Lead Oxide, 00-035-1482
20
30
40
50
60
70
80
Position [°2Theta] (Copper (Cu))
