# Supplementary material for: Hydrothermally synthesized PZT film grown in highly concentrated KOH solution with large electromechanical coupling coefficient for resonator
Source: R Soc Open Sci. 2017 Dec 20;4(12):171363. doi: 10.1098/rsos.171363 (PMC5750027; doi:10.1098/rsos.171363)

Name and formula

Reference code:	00-033-1381
Compound name:	Titanium Oxide
Empirical formula:	O_2Ti
Chemical formula:	TiO_2

Crystallographic parameters

Crystal system:	Hexagonal
Space group:	P63E
Space group number:	173
a (A):	9.2200
b (A):	9.2200
c (A):	5.6850
Alpha (°):	90.0000
Beta (°):	90.0000
Gamma (°):	120.0000
Volume of cell (10^6 pm^3):	418.53
Z:	16.00
RIR:	-

Subfiles and quality

Subfiles:	Alloy, metal or intermetalic
	Excipient
	Inorganic
	Pharmaceutical
Quality:	Indexed (I)

Comments

Creation Date:	5/20/1982
Modification Date:	1/11/2013
General Comments:	Z-tentative assignment, made on the basis of volume changes. On release of pressure, transition to a-"Pb O2" form results in 8.5% volume change
Sample Preparation:	X-ray diffraction patterns obtained in situ in a diamond-anvil pressure cell after laser heating and quenching
Unit Cell:	a and c measured at room temperature, approximately 250 kbar.

References

Primary reference: Liu, L., *Science*, **199**, 422, (1978)

Peak list

No.	h	k	l	d [A]	2Theta[deg]	I [%]
1	0	0	2	2.83000	31.589	5.0
2	2	1	1	2.66700	33.575	100.0
3	3	0	0	2.66700	33.575	100.0
4	3	0	1	2.41000	37.281	10.0
5	2	2	0	2.30200	39.099	20.0
6	4	1	1	1.66400	55.151	30.0
7	2	1	3	1.60900	57.207	5.0
8	4	2	0	1.51000	61.345	5.0
9	0	0	4	1.42000	65.703	10.0
10	4	0	3	1.37900	67.917	5.0

Stick Pattern

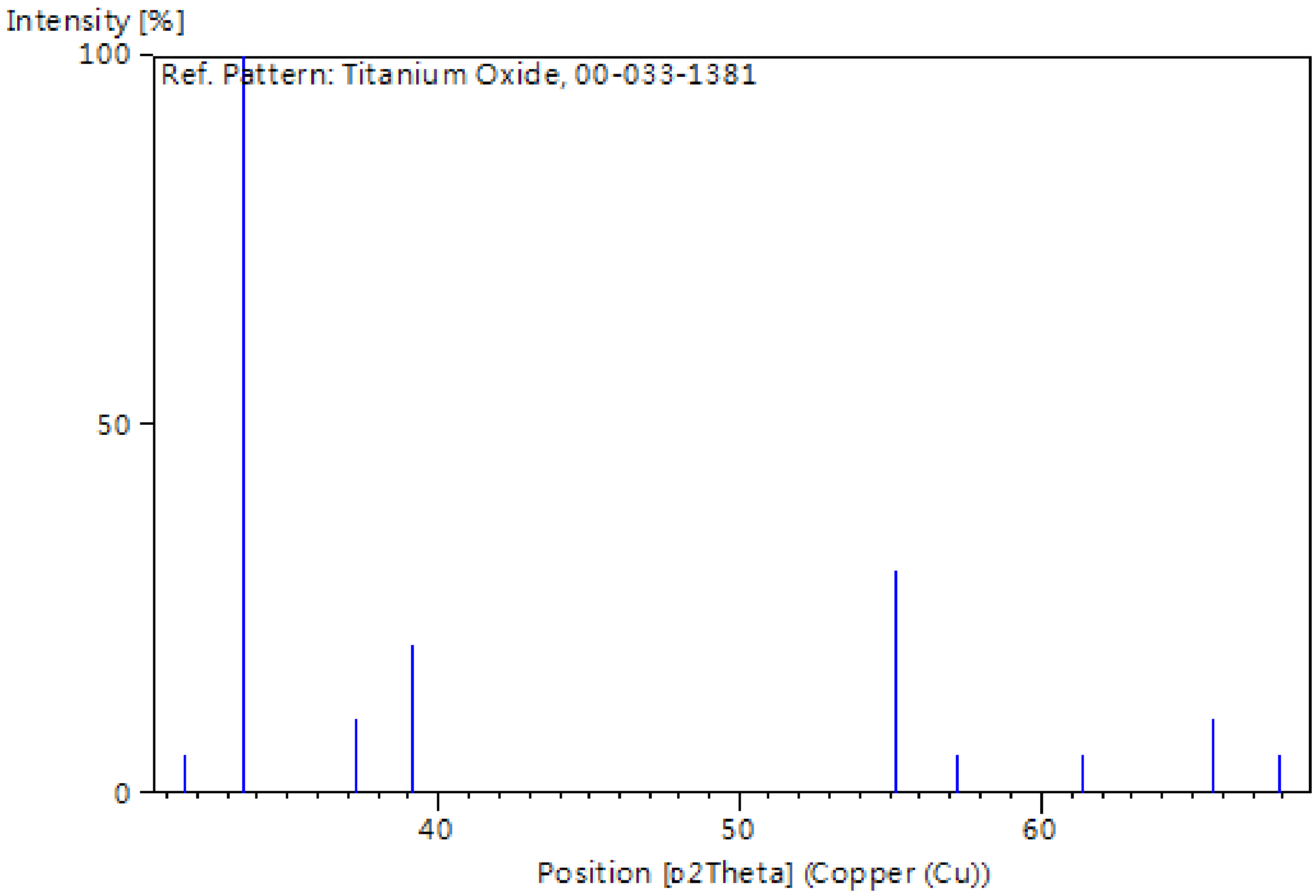

Supplement: XRD code dataset [file rsos171363supp4.pdf]
