# Supplementary material for: Hydrothermally synthesized PZT film grown in highly concentrated KOH solution with large electromechanical coupling coefficient for resonator
Source: R Soc Open Sci. 2017 Dec 20;4(12):171363. doi: 10.1098/rsos.171363 (PMC5750027; doi:10.1098/rsos.171363)

Name and formula

Reference code:	01-076-0321
Compound name:	Titanium Oxide
Common name:	Rutile, syn
Empirical formula:	O_2Ti
Chemical formula:	TiO_2

Crystallographic parameters

Crystal system:	Tetragonal
Space group:	P42/mnm
Space group number:	136
a (A):	4.6252
b (A):	4.6252
c (A):	2.9878
Alpha (°):	90.0000
Beta (°):	90.0000
Gamma (°):	90.0000
Volume of cell (10^6 pm^3):	63.92
Z:	2.00
RIR:	3.34

Status, subfiles and quality

Status:	Diffraction data collected at non ambient temperature
	Alternate Pattern
Subfiles:	Alloy, metal or intermetalic
	Common Phase
	Excipient
	Forensic
	ICSD Pattern
	Inorganic
	Mineral
	Pharmaceutical
Quality:	Star (S)

Comments

ANX:	AX2
ICSD collection code:	33841
Creation Date:	7/26/2010
Modification Date:	1/17/2013

ANX: AX2
Analysis: O2 Ti1
Formula from original source: Ti O2
ICSD Collection Code: 33841
Calculated Pattern Original Remarks: Stable from 800 to 1800 K (2nd ref., Tomaszewski), 300-800 K: Pbca. Temperature of Data Collection: 1163 K. Wyckoff Sequence: f a(P42/MNM). Unit Cell Data Source: Single Crystal.

References

Primary reference: *Calculated from ICSD using POWD-12++*, (2004)
Structure: Sugiyama, K., Takeuchi, Y., *Z. Kristallogr.*, **194**, 305, (1991)

Peak list

No.	h	k	l	d [A]	2Theta[deg]	I [%]
1	1	1	0	3.27050	27.246	100.0
2	1	0	1	2.50970	35.749	45.4
3	2	0	0	2.31260	38.913	6.5
4	1	1	1	2.20590	40.877	17.5
5	2	1	0	2.06840	43.729	6.3
6	2	1	1	1.70070	53.864	46.4
7	2	2	0	1.63520	56.208	12.4
8	0	0	2	1.49390	62.079	6.4
9	3	1	0	1.46260	63.561	5.6
10	2	2	1	1.43450	64.957	0.4
11	3	0	1	1.37010	68.419	13.4
12	1	1	2	1.35880	69.068	7.0
13	3	1	1	1.31370	71.798	0.7
14	3	2	0	1.28280	73.809	0.2
15	2	0	2	1.25480	75.742	1.4
16	2	1	2	1.21110	78.993	0.8
17	3	2	1	1.17880	81.606	2.1
18	4	0	0	1.15630	83.545	1.4
19	4	1	0	1.12180	86.733	0.5
20	2	2	2	1.10290	88.603	3.6
21	3	3	0	1.09020	89.913	1.7
22	4	1	1	1.05020	94.358	2.7
23	3	1	2	1.04510	94.963	2.4
24	4	2	0	1.03420	96.288	1.3
25	3	3	1	1.02410	97.558	0.1
26	4	2	1	0.97730	104.034	0.3
27	3	2	2	0.97360	104.593	1.0
28	1	0	3	0.97360	104.593	1.0
29	1	1	3	0.95270	107.908	0.3
30	4	3	0	0.92500	112.766	0.1
31	4	0	2	0.91440	114.791	1.1
32	5	1	0	0.90710	116.247	1.1
33	4	1	2	0.89730	118.289	3.0
34	2	1	3	0.89730	118.289	3.0
35	5	0	1	0.88370	121.308	2.2
36	3	3	2	0.88060	122.030	1.6
37	5	1	1	0.86800	125.107	0.1
38	4	2	2	0.85030	129.894	1.5
39	2	2	3	0.85030	129.894	1.5
40	3	0	3	0.83660	134.072	1.7
41	5	2	1	0.82540	137.893	2.4

42	3	1	3	0.82320	138.696	0.1
43	4	4	0	0.81760	140.831	0.2

Stick Pattern

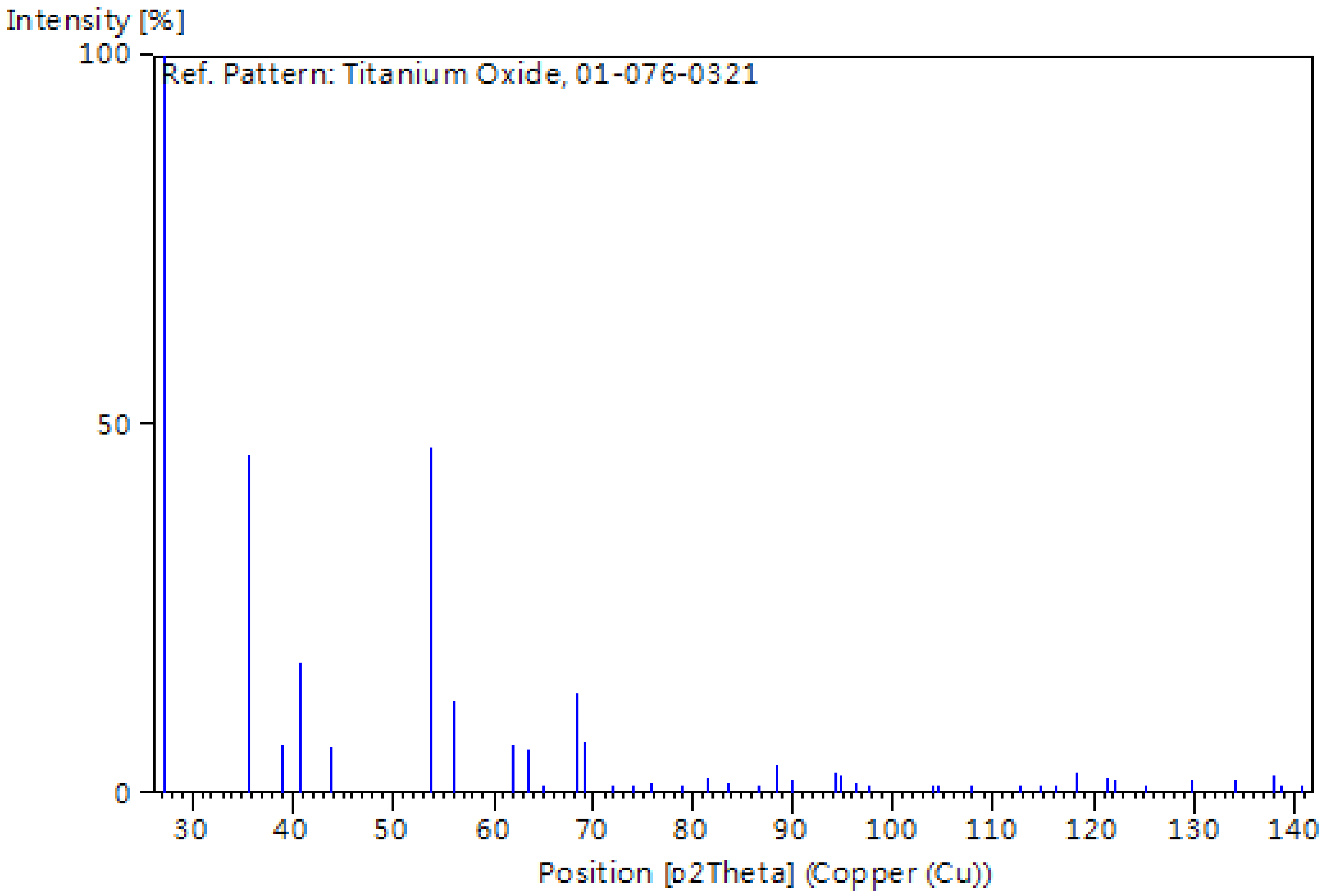

Supplement: XRD code dataset [file rsos171363supp5.pdf]
