# Supplementary material for: Hydrothermally synthesized PZT film grown in highly concentrated KOH solution with large electromechanical coupling coefficient for resonator
Source: R Soc Open Sci. 2017 Dec 20;4(12):171363. doi: 10.1098/rsos.171363 (PMC5750027; doi:10.1098/rsos.171363)

Name and formula

Reference code:	01-070-4261
Compound name:	Lead Zirconium Titanium Oxide
Empirical formula:	$O_3PbTi_{0.7}Zr_{0.3}$
Chemical formula:	$Pb\ (\ Zr_{0.3}Ti_{0.7}\ )\ O_3$

Crystallographic parameters

Crystal system:	Tetragonal
Space group:	P4mm
Space group number:	99
a (A):	3.9862
b (A):	3.9862
c (A):	4.1331
Alpha (°):	90.0000
Beta (°):	90.0000
Gamma (°):	90.0000
Volume of cell (10^6 pm^3):	65.67
Z:	1.00
RIR:	12.76

Subfiles and quality

Subfiles:	ICSD Pattern
	Inorganic
Quality:	Star (S)

Comments

ANX:	ABX3
ICSD collection code:	90696
Creation Date:	7/27/2010
Modification Date:	1/17/2013
ANX:	ABX3
Analysis:	O3 Pb1 Ti0.7 Zr0.3
Formula from original source:	Pb (Zr0.3 Ti0.7) O3
ICSD Collection Code:	90696
Wyckoff Sequence:	c b2 a(P4MM)
Unit Cell Data Source:	Powder Diffraction.

References

Primary reference: *Calculated from ICSD using POWD-12++*
Structure: Joseph, J., Vimala, T.M., Sivasubramanian, V., Murthy, V.R.K., *J. Mater. Sci.*, **35**, 1571, (2000)

Peak list

No.	h	k	l	d [A]	2Theta[deg]	I [%]
1	0	0	1	4.13310	21.482	15.1
2	1	0	0	3.98620	22.284	24.3
3	1	0	1	2.86920	31.147	100.0
4	1	1	0	2.81870	31.719	46.5
5	1	1	1	2.32870	38.633	25.7
6	0	0	2	2.06660	43.769	10.9
7	2	0	0	1.99310	45.472	24.9
8	1	0	2	1.83470	49.650	8.4
9	2	0	1	1.79530	50.816	6.0
10	2	1	0	1.78270	51.201	5.4
11	1	1	2	1.66660	55.058	18.3
12	2	1	1	1.63690	56.145	33.2
13	2	0	2	1.43460	64.952	11.2
14	2	2	0	1.40930	66.266	6.4
15	0	0	3	1.37770	67.990	0.8
16	2	1	2	1.34980	69.595	5.2
17	2	2	1	1.33390	70.547	2.0
18	3	0	0	1.32870	70.864	1.0
19	1	0	3	1.30210	72.539	7.4
20	3	0	1	1.26500	75.025	6.2
21	3	1	0	1.26060	75.332	6.0
22	1	1	3	1.23780	76.971	1.4
23	3	1	1	1.20570	79.417	4.1
24	2	2	2	1.16440	82.835	4.9
25	2	0	3	1.13330	85.640	1.5
26	3	0	2	1.11760	87.141	1.2
27	3	2	0	1.10560	88.330	0.9
28	2	1	3	1.09010	89.923	7.4
29	3	1	2	1.07610	91.422	6.8
30	3	2	1	1.06800	92.316	6.6
31	0	0	4	1.03330	96.400	0.6
32	1	0	4	1.00020	100.734	1.2
33	4	0	0	0.99660	101.235	1.7
34	2	2	3	0.98520	102.865	1.0
35	3	2	2	0.97480	104.411	1.6
36	1	1	4	0.97010	105.130	2.6
37	4	0	1	0.96880	105.331	0.8
38	4	1	0	0.96680	105.642	0.7
39	3	0	3	0.95640	107.301	2.5
40	4	1	1	0.94140	109.820	4.7
41	3	3	0	0.93960	110.133	1.3
42	3	1	3	0.93000	111.845	1.2
43	2	0	4	0.91730	114.227	1.8
44	3	3	1	0.91620	114.440	0.9
45	4	0	2	0.89760	118.225	2.3
46	2	1	4	0.89400	119.001	1.8
47	4	2	0	0.89130	119.593	2.7
48	4	1	2	0.87570	123.198	1.3
49	4	2	1	0.87130	124.277	1.1
50	3	2	3	0.86230	126.584	4.3
51	3	3	2	0.85530	128.479	2.1
52	2	2	4	0.83330	135.155	1.6

53	0	0	5	0.82660	137.463	0.2
54	4	2	2	0.81850	140.478	4.3
55	3	0	4	0.81570	141.588	0.8
56	1	0	5	0.80940	144.237	1.9
57	4	0	3	0.80750	145.082	0.7
58	3	1	4	0.79910	149.141	4.1

Stick Pattern

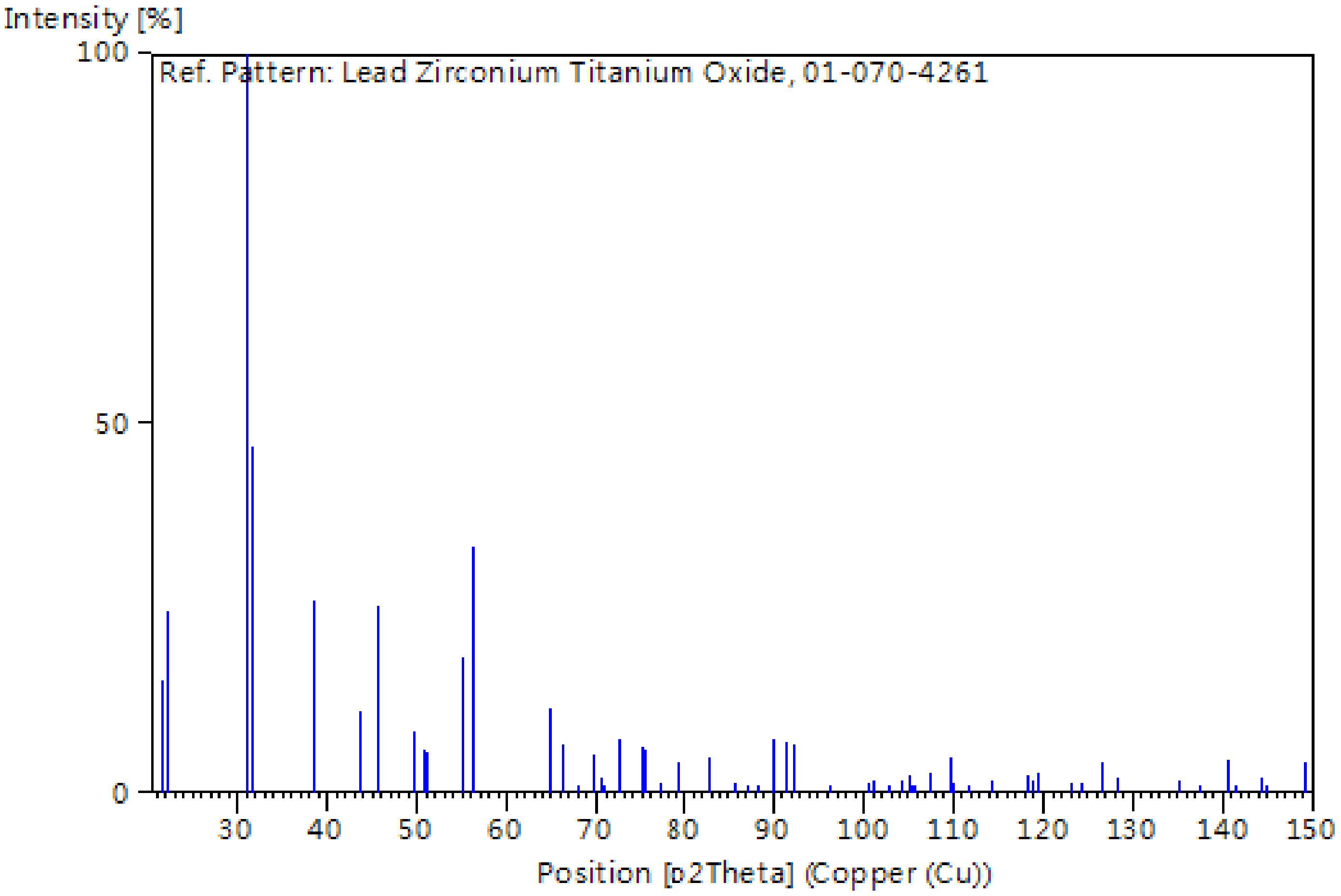

Supplement: XRD code dataset [file rsos171363supp6.pdf]
