# Supplementary material for: Hydrothermally synthesized PZT film grown in highly concentrated KOH solution with large electromechanical coupling coefficient for resonator
Source: R Soc Open Sci. 2017 Dec 20;4(12):171363. doi: 10.1098/rsos.171363 (PMC5750027; doi:10.1098/rsos.171363)

Name and formula

Reference code:	01-070-0743
Compound name:	Lead Titanium Zirconium Oxide
Empirical formula:	$O_3PbTi_{0.1}Zr_{0.9}$
Chemical formula:	$Pb (Zr_{0.9}Ti_{0.1}) O_3$

Crystallographic parameters

Crystal system:	Rhombohedral
Space group:	R3c
Space group number:	161
a (A):	5.8564
b (A):	5.8564
c (A):	14.3951
Alpha (°):	90.0000
Beta (°):	90.0000
Gamma (°):	120.0000
Volume of cell (10^6 pm^3):	427.57
Z:	6.00
RIR:	9.09

Status, subfiles and quality

Status:	Diffraction data collected at non ambient temperature<br>Alternate Pattern
Subfiles:	Ceramic<br>ICSD Pattern<br>Inorganic
Quality:	Indexed (I)

Comments

ANX:	ABX3
ICSD collection code:	1608
Creation Date:	11/15/2010
Modification Date:	1/17/2013
ANX:	ABX3
ICSD Collection Code:	1608
Calculated Pattern Original Remarks:	Pseudo-cubic (alpha= 89.867 grd), R(profile)= 0.1628, becomes cubic above 523 K (Pm3-m), cf. 1609
Structures:	(the sample crystallizes in the space group R3m, but is described in the space group R3C)

Temperature of Data Collection: 508 K
Minor Warning: No e.s.d reported/abstracted on the cell dimension
Unit Cell Data Source: Powder Diffraction.

References

Primary reference: *Calculated from ICSD using POWD-12++*, (1997)
Structure: Glazer, A.M., Mabud, S.A., Clarke, R., *Acta Crystallogr., Sec. B: Struct. Crystallogr. Cryst. Chem.*, **34**, 1060, (1978)

Peak list

No.	h	k	l	d [A]	2Theta[deg]	I [%]
1	0	1	2	4.14590	21.415	22.1
2	1	0	4	2.93500	30.431	100.0
3	1	1	0	2.92820	30.504	96.8
4	0	0	6	2.39920	37.455	6.3
5	2	0	2	2.39180	37.575	11.3
6	0	2	4	2.07290	43.629	38.8
7	1	1	6	1.85580	49.048	5.6
8	0	1	8	1.69580	54.032	15.3
9	2	1	4	1.69190	54.167	28.7
10	3	0	0	1.69190	54.167	28.7
11	1	2	5	1.59560	57.732	0.1
12	2	0	8	1.46750	63.324	8.8
13	2	2	0	1.46410	63.488	8.0
14	1	0	10	1.38480	67.594	1.7
15	0	3	6	1.38200	67.750	1.0
16	3	1	2	1.38200	67.750	1.0
17	1	2	8	1.31200	71.906	7.5
18	1	3	4	1.31010	72.026	6.8
19	0	2	10	1.25190	75.948	1.0
20	0	4	2	1.24980	76.099	0.7
21	2	2	6	1.24980	76.099	0.7
22	0	0	12	1.19960	79.902	1.1
23	4	0	4	1.19590	80.199	2.6
24	2	1	10	1.15110	84.009	0.9
25	1	1	12	1.11000	87.889	3.7
26	3	1	8	1.10820	88.069	3.5
27	4	1	0	1.10710	88.179	4.7
28	3	2	4	1.10710	88.179	4.7
29	1	2	11	1.08080	90.912	0.1
30	4	1	3	1.07840	91.172	0.1
31	0	4	8	1.03650	96.005	1.3
32	0	1	14	1.00770	99.710	0.6
33	1	3	10	1.00610	99.926	0.4
34	5	0	2	1.00500	100.075	0.3
35	1	4	6	1.00500	100.075	0.3
36	0	3	12	0.97830	103.884	1.9
37	2	3	8	0.97710	104.064	1.9
38	0	5	4	0.97630	104.184	1.6
39	3	3	0	0.97630	104.184	1.6
40	3	1	11	0.95810	107.025	0.1
41	2	0	14	0.95290	107.875	0.4
42	4	0	10	0.95150	108.107	0.1
43	4	2	2	0.95010	108.340	0.1
44	2	2	12	0.92790	112.229	1.6
45	2	4	4	0.92620	112.543	1.5

46	5	1	1	0.90910	115.843	0.1
47	4	2	5	0.90910	115.843	0.1
48	1	2	14	0.90610	116.451	0.6
49	3	2	10	0.90490	116.697	0.3
50	1	5	2	0.90410	116.861	0.2
51	3	3	6	0.90410	116.861	0.2
52	1	0	16	0.88590	120.804	0.8
53	5	0	8	0.88310	121.447	1.1
54	5	1	4	0.88310	121.447	1.1
55	0	2	16	0.84790	130.592	0.7
56	4	2	8	0.84590	131.184	1.1
57	6	0	0	0.84530	131.364	0.7
58	4	3	1	0.83240	135.456	0.1
59	3	1	14	0.83010	136.238	0.4
60	0	5	10	0.82920	136.549	0.1
61	3	4	2	0.82830	136.862	0.1
62	2	1	16	0.81450	142.076	1.1
63	4	1	12	0.81340	142.529	1.8
64	5	2	0	0.81230	142.990	1.7
65	4	3	4	0.81230	142.990	1.7
66	2	2	15	0.80260	147.380	0.1
67	0	0	18	0.79970	148.831	0.2
68	0	4	14	0.79860	149.402	0.1
69	2	4	10	0.79780	149.825	0.1

Stick Pattern

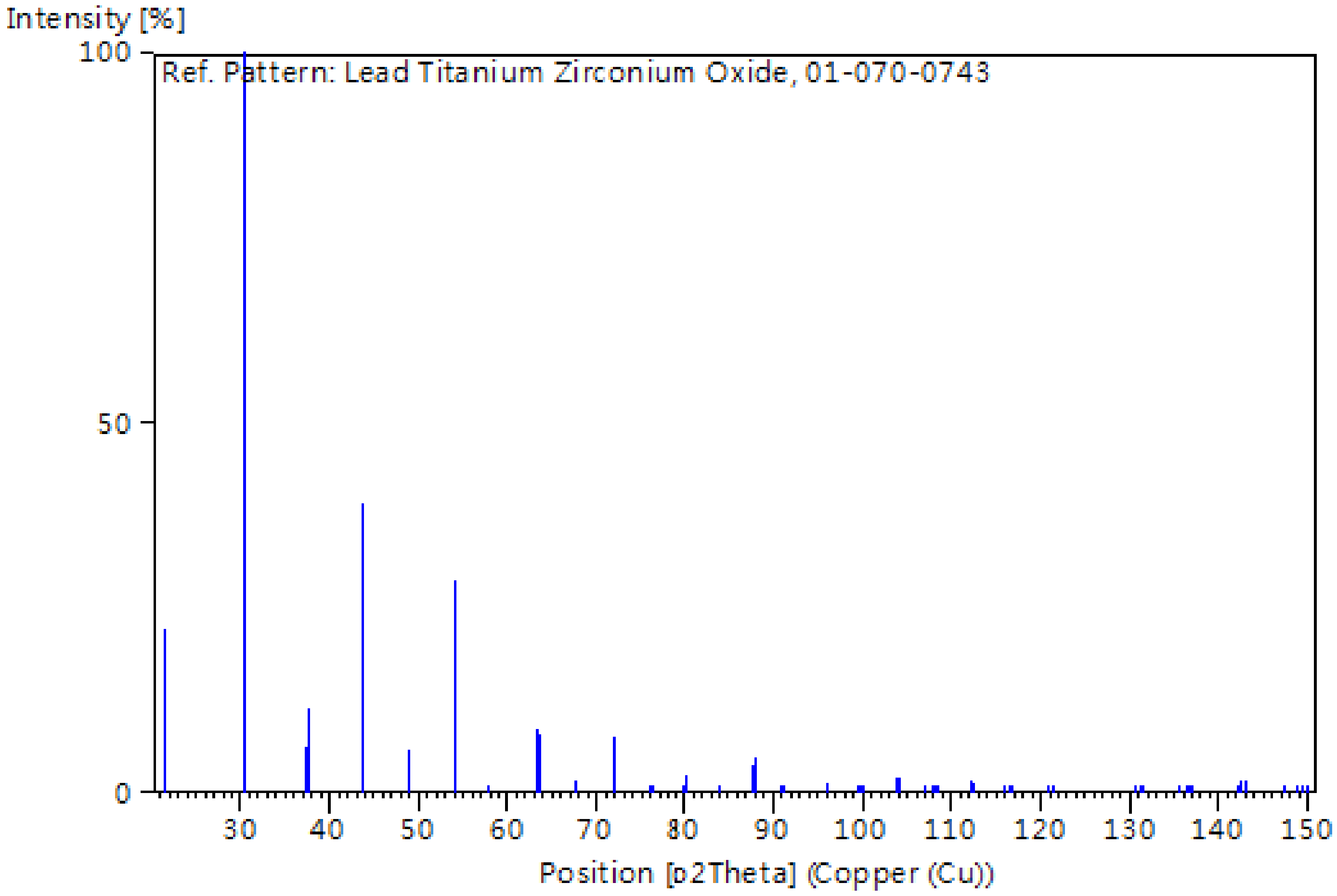

Supplement: XRD code dataset [file rsos171363supp7.pdf]
