# Supplementary material for: Hydrothermally synthesized PZT film grown in highly concentrated KOH solution with large electromechanical coupling coefficient for resonator
Source: R Soc Open Sci. 2017 Dec 20;4(12):171363. doi: 10.1098/rsos.171363 (PMC5750027; doi:10.1098/rsos.171363)

Name and formula

Reference code:	03-065-3362
Compound name:	Titanium
Empirical formula:	Ti
Chemical formula:	Ti

Crystallographic parameters

Crystal system:	Hexagonal
Space group:	P63/mmc
Space group number:	194
a (A):	2.9504
b (A):	2.9504
c (A):	4.6833
Alpha (°):	90.0000
Beta (°):	90.0000
Gamma (°):	120.0000
Volume of cell (10^6 pm^3):	35.31
Z:	2.00
RIR:	6.77

Subfiles and quality

Subfiles:	Alloy, metal or intermetalic
	Common Phase
	Explosive
	Forensic
	Inorganic
	NIST Pattern
Quality:	Indexed (I)

Comments

Creation Date:	2/11/2005
Modification Date:	1/17/2013
Additional Patterns:	See PDF 01-089-2762
NIST M&A collection code:	N AL4838 7420
Physical property:	Hydrogen storage materials
Temperature Factor:	IB=Ti
Minor Warning:	No e.s.d reported/abstracted on the cell dimension. No Rfactor reported/abstracted.

References

Primary reference: *Calculated from NIST using POWD-12++*
Structure: H.Clark, *J. Met.*, **1**, 588, (1949)

Peak list

No.	h	k	l	d [A]	2Theta[deg]	I [%]
1	1	0	0	2.55510	35.092	25.7
2	0	0	2	2.34160	38.412	25.8
3	1	0	1	2.24300	40.171	100.0
4	1	0	2	1.72630	53.002	12.4
5	1	1	0	1.47520	62.955	12.6
6	1	0	3	1.33210	70.656	12.1
7	2	0	0	1.27760	74.160	1.7
8	1	1	2	1.24820	76.214	12.1
9	2	0	1	1.23250	77.363	8.7
10	0	0	4	1.17080	82.284	1.6
11	2	0	2	1.12150	86.762	1.9
12	1	0	4	1.06440	92.721	1.6
13	2	0	3	0.98870	102.357	3.7
14	2	1	0	0.96570	105.814	1.2
15	2	1	1	0.94580	109.065	6.6
16	1	1	4	0.91710	114.266	4.2
17	2	1	2	0.89280	119.263	2.0
18	1	0	5	0.87940	122.312	2.9
19	2	0	4	0.86320	126.347	0.9
20	3	0	0	0.85170	129.492	1.9
21	2	1	3	0.82130	139.405	5.5
22	3	0	2	0.80040	148.474	3.7

Stick Pattern

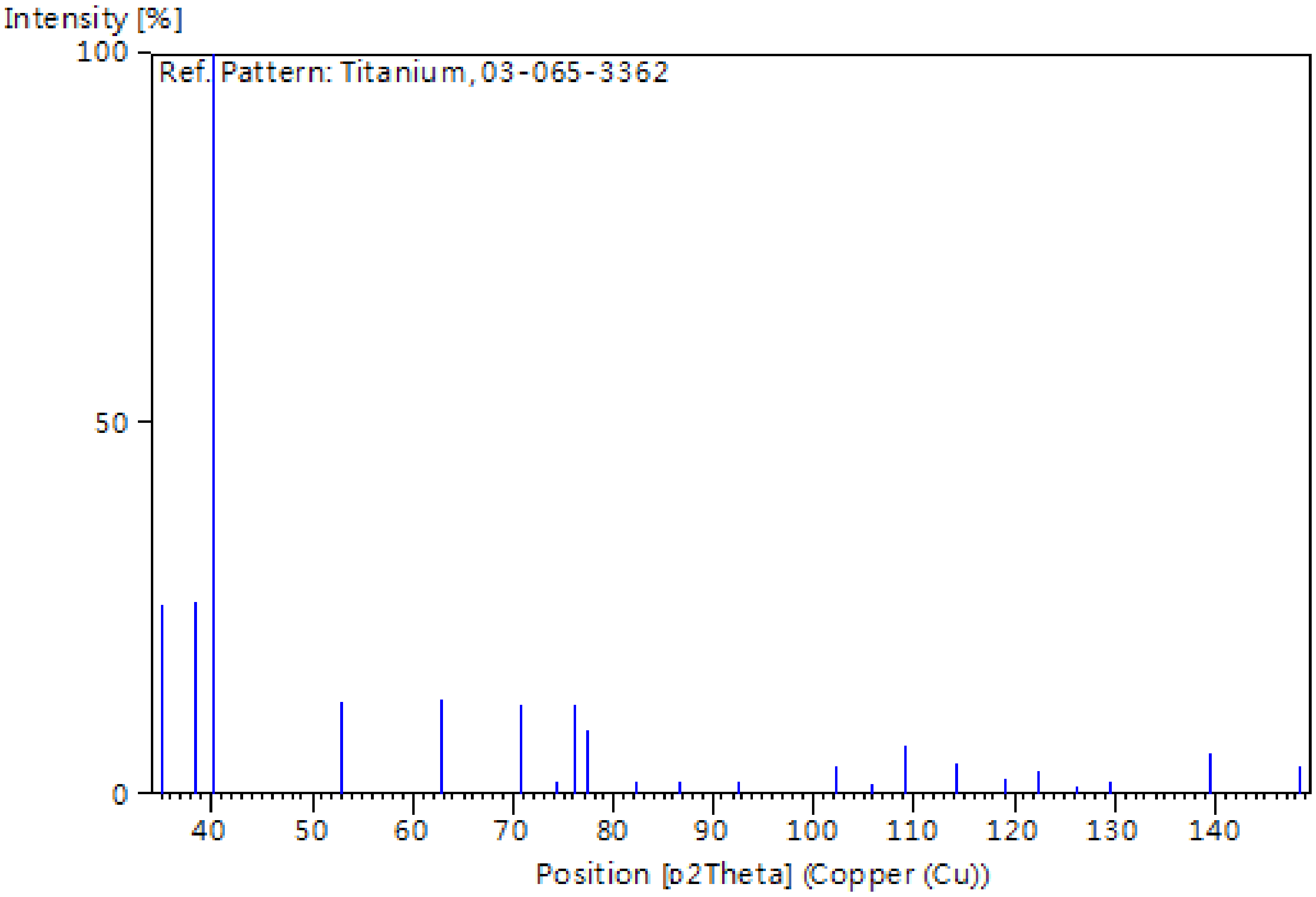

Supplement: XRD code dataset [file rsos171363supp8.pdf]
