# Supplementary material for: Hydrothermally synthesized PZT film grown in highly concentrated KOH solution with large electromechanical coupling coefficient for resonator
Source: R Soc Open Sci. 2017 Dec 20;4(12):171363. doi: 10.1098/rsos.171363 (PMC5750027; doi:10.1098/rsos.171363)
Supplement: XRD code dataset [file rsos171363supp9.pdf]

Name and formula

Reference code:	01-089-2959
Compound name:	Titanium
Empirical formula:	Ti
Chemical formula:	Ti

Crystallographic parameters

Crystal system:	Hexagonal
Space group:	P63/mmc
Space group number:	194
a (A):	2.9510
b (A):	2.9510
c (A):	4.6820
Alpha (°):	90.0000
Beta (°):	90.0000
Gamma (°):	120.0000
Volume of cell (10^6 pm^3):	35.31
Z:	2.00
RIR:	6.44

Status, subfiles and quality

Status:	Alternate Pattern
Subfiles:	Alloy, metal or intermetalic
	Common Phase
	Explosive
	Forensic
	ICSD Pattern
	Inorganic
Quality:	Indexed (I)

Comments

ANX:	N
ICSD collection code:	43614
Creation Date:	7/27/2010
Modification Date:	1/17/2013
ANX:	N
Analysis:	Ti1
Formula from original source:	Ti
ICSD Collection Code:	43614

Physical property: Hydrogen storage materials
Calculated Pattern Original Remarks: Stable up to 1153 K (2nd ref., Tomaszewski), above Im3-m, m.p. 1940 K
Cell at 973 K: 2.971, 4.721
Minor Warning: No e.s.d reported/abstracted on the cell dimension. No R factors reported/abstracted
Wyckoff Sequence: c(P63/MMC)
Unit Cell Data Source: Single Crystal.

References

Primary reference: *Calculated from ICSD using POWD-12++*
Structure: Wasilewski, R.J., *Trans. Met. Soc. AIME*, **221**, 1231, (1961)

Peak list

No.	h	k	l	d [A]	2Theta[deg]	I [%]
1	1	0	0	2.55560	35.085	25.5
2	0	0	2	2.34100	38.422	25.8
3	1	0	1	2.24320	40.168	100.0
4	1	0	2	1.72620	53.005	12.1
5	1	1	0	1.47550	62.941	12.3
6	1	0	3	1.33200	70.663	11.3
7	2	0	0	1.27780	74.146	1.6
8	1	1	2	1.24820	76.214	11.3
9	2	0	1	1.23270	77.348	8.0
10	0	0	4	1.17050	82.309	1.4
11	2	0	2	1.12160	86.753	1.7
12	1	0	4	1.06420	92.743	1.4
13	2	0	3	0.98870	102.357	3.1
14	2	1	0	0.96590	105.783	1.0
15	2	1	1	0.94600	109.031	5.5
16	1	1	4	0.91700	114.285	3.4
17	2	1	2	0.89290	119.241	1.6
18	1	0	5	0.87920	122.360	2.3
19	2	0	4	0.86310	126.373	0.7
20	3	0	0	0.85190	129.435	1.5
21	2	1	3	0.82140	139.367	4.2
22	3	0	2	0.80050	148.423	2.7

Stick Pattern

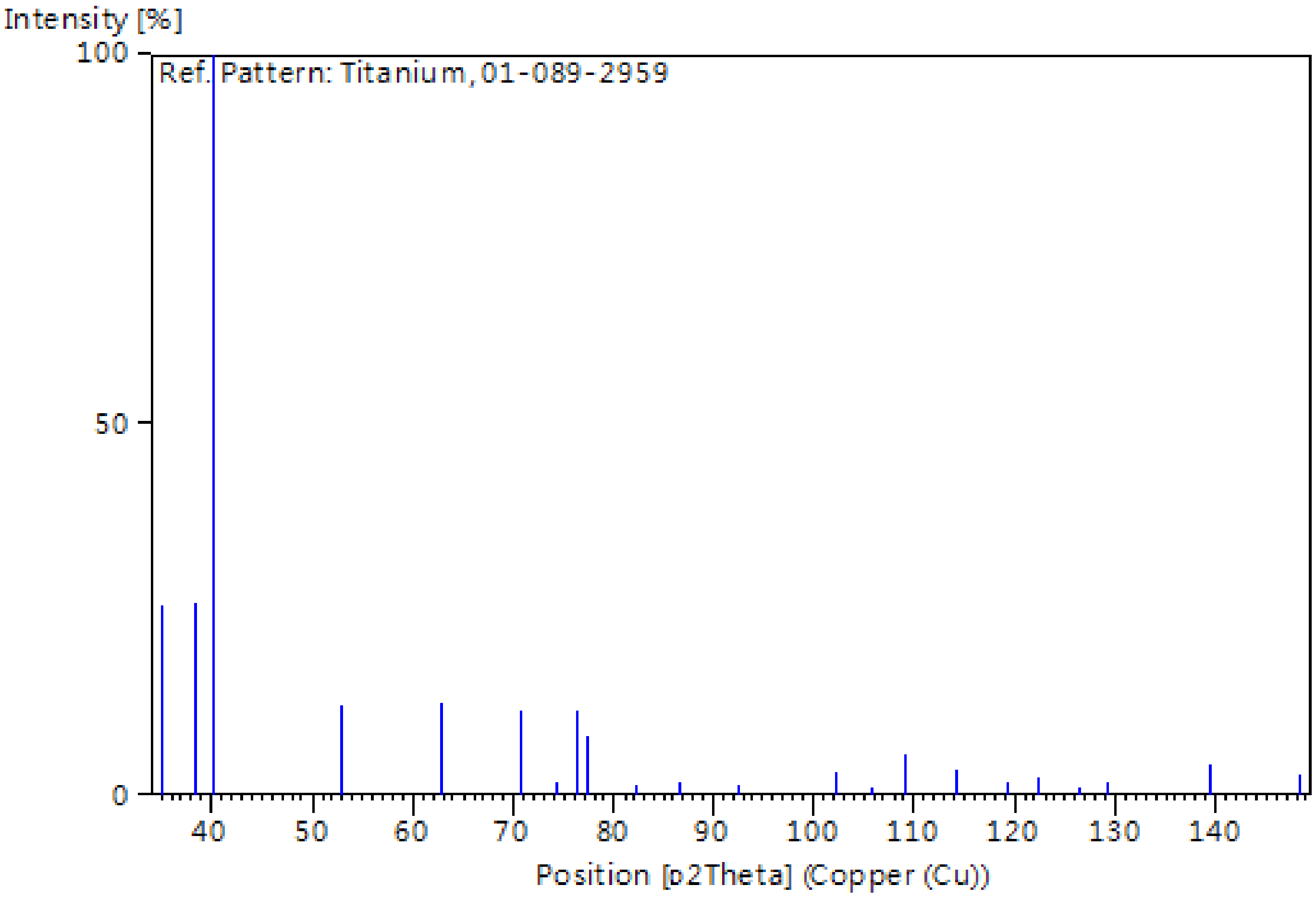
Intensity [%]
100
50
0
Ref. Pattern: Titanium, 01-089-2959
40
50
60
70
80
90
100
110
120
130
140
Position [°2Theta] (Copper (Cu))
