# Supplementary material for: Hydrothermally synthesized PZT film grown in highly concentrated KOH solution with large electromechanical coupling coefficient for resonator
Source: R Soc Open Sci. 2017 Dec 20;4(12):171363. doi: 10.1098/rsos.171363 (PMC5750027; doi:10.1098/rsos.171363)

Name and formula

Reference code:	00-044-1288
Compound name:	Titanium
Common name:	a-Ti
Empirical formula:	Ti
Chemical formula:	Ti

Crystallographic parameters

Crystal system:	Cubic
Space group:	Im-3m
Space group number:	229
a (A):	3.3065
b (A):	3.3065
c (A):	3.3065
Alpha (°):	90.0000
Beta (°):	90.0000
Gamma (°):	90.0000
Calculated density (g/cm^3):	4.40
Volume of cell (10^6 pm^3):	36.15
Z:	2.00
RIR:	8.68

Subfiles and quality

Subfiles:	Alloy, metal or intermetalic
	Explosive
	Inorganic
Quality:	Calculated (C)

Comments

Creation Date:	7/26/1993
Modification Date:	1/11/2013
General Comments:	High temperature phase, stable above 1153 K
Physical property:	Hydrogen storage materials.

References

Primary reference:	Calvert, L., Lakes Entrance, Victoria, Australia., *Private Communication*, (1993)
Unit cell:	Eppelsheimer, D., Perman, R., *Nature (London)*, **166**, 960, (1950)

Peak list

No.	h	k	l	d [A]	2Theta[deg]	I [%]
1	1	1	0	2.33750	38.482	100.0
2	2	0	0	1.65320	55.543	12.0
3	2	1	1	1.34960	69.607	17.0
4	2	2	0	1.16890	82.447	4.0
5	3	1	0	1.04540	94.927	5.0
6	2	2	2	0.95440	107.628	1.0
7	3	2	1	0.88370	121.308	6.0
8	4	0	0	0.82660	137.463	1.0
9	4	1	1	0.77930	162.568	4.0

Stick Pattern

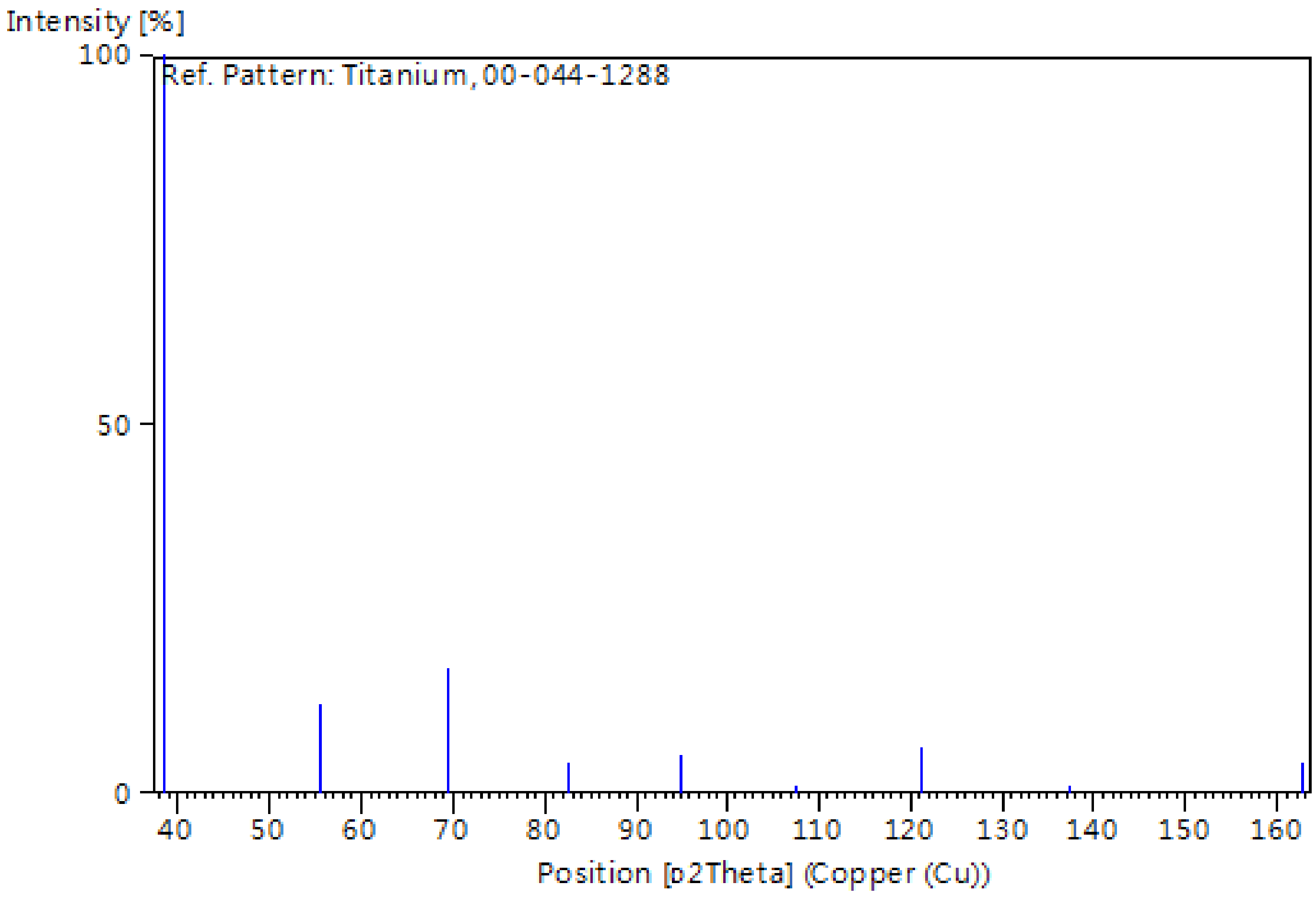

Supplement: XRD code dataset [file rsos171363supp10.pdf]
