# Supplementary material for: Hydrothermally synthesized PZT film grown in highly concentrated KOH solution with large electromechanical coupling coefficient for resonator
Source: R Soc Open Sci. 2017 Dec 20;4(12):171363. doi: 10.1098/rsos.171363 (PMC5750027; doi:10.1098/rsos.171363)

Name and formula

Reference code:	01-072-7166
Compound name:	Lead Zirconium Titanium Oxide
Empirical formula:	$O_3PbTi_{0.485}Zr_{0.515}$
Chemical formula:	$Pb\ (\ Zr_{0.515}Ti_{0.485}\ )\ O_3$

Crystallographic parameters

Crystal system:	Tetragonal
Space group:	P4mm
Space group number:	99
a (A):	4.0174
b (A):	4.0174
c (A):	4.1420
Alpha (°):	90.0000
Beta (°):	90.0000
Gamma (°):	90.0000
Volume of cell (10^6 pm^3):	66.85
Z:	1.00
RIR:	11.16

Subfiles and quality

Subfiles:	ICSD Pattern
	Inorganic
Quality:	Star (S)

Comments

ANX:	ABX3
ICSD collection code:	97057
Creation Date:	7/27/2010
Modification Date:	1/17/2013
ANX:	ABX3
Analysis:	O3 Pb1 Ti0.485 Zr0.515
Formula from original source:	Pb (Zr0.515 Ti0.485) O3
ICSD Collection Code:	97057
Wyckoff Sequence:	c b2 a(P4MM)
Unit Cell Data Source:	Powder Diffraction.

References

Primary reference: *Calculated from ICSD using POWD-12++*
Structure: Pandey, D., Mishra, S.K., Ranjan, R., Ragini, *J. Appl. Phys.* , **92**, 3266, (2002)

Peak list

No.	h	k	l	d [A]	2Theta[deg]	I [%]
1	0	0	1	4.14200	21.436	15.4
2	1	0	0	4.01740	22.109	20.0
3	1	0	1	2.88380	30.985	100.0
4	1	1	0	2.84070	31.467	46.7
5	1	1	1	2.34270	38.393	20.4
6	0	0	2	2.07100	43.671	10.4
7	2	0	0	2.00870	45.099	22.2
8	1	0	2	1.84080	49.475	6.3
9	2	0	1	1.80740	50.452	3.6
10	2	1	0	1.79660	50.777	2.5
11	1	1	2	1.67350	54.812	14.6
12	2	1	1	1.64830	55.722	24.2
13	2	0	2	1.44190	64.583	7.8
14	2	2	0	1.42040	65.682	4.3
15	0	0	3	1.38070	67.822	0.7
16	2	1	2	1.35710	69.167	2.6
17	2	2	1	1.34360	69.963	0.7
18	3	0	0	1.33910	70.232	0.3
19	1	0	3	1.30570	72.307	4.1
20	3	0	1	1.27420	74.391	3.3
21	3	1	0	1.27040	74.651	3.1
22	1	1	3	1.24180	76.677	0.8
23	3	1	1	1.21460	78.721	1.3
24	2	2	2	1.17140	82.233	2.5
25	2	0	3	1.13780	85.221	0.9
26	3	0	2	1.12450	86.474	0.4
27	3	2	0	1.11420	87.474	0.1
28	2	1	3	1.09480	89.433	3.0
29	3	1	2	1.08290	90.687	2.8
30	3	2	1	1.07600	91.433	2.5
31	0	0	4	1.03550	96.128	0.2
32	4	0	0	1.00440	100.157	0.6
33	1	0	4	1.00270	100.390	0.6
34	2	2	3	0.99000	102.170	0.4
35	3	2	2	0.98120	103.452	0.3
36	4	0	1	0.97610	104.214	0.1
37	1	1	4	0.97290	104.700	0.9
38	3	0	3	0.96130	106.511	0.7
39	4	1	1	0.94850	108.608	1.3
40	3	3	0	0.94690	108.878	0.3
41	3	1	3	0.93490	110.962	0.3
42	3	3	1	0.92310	113.122	0.1
43	2	0	4	0.92040	113.632	0.5
44	4	0	2	0.90370	116.944	0.6
45	4	2	0	0.89830	118.076	0.8
46	2	1	4	0.89720	118.311	0.7
47	4	1	2	0.88170	121.772	0.2
48	4	2	1	0.87790	122.669	0.1
49	3	2	3	0.86710	125.337	0.9
50	3	3	2	0.86120	126.876	0.4
51	2	2	4	0.83670	134.040	0.3
52	0	0	5	0.82840	136.827	0.1

53	4	2	2	0.82410	138.365	0.8
54	3	0	4	0.81920	140.207	0.2
55	4	0	3	0.81220	143.032	0.2
56	1	0	5	0.81130	143.414	0.4
57	3	1	4	0.80260	147.380	0.8

Stick Pattern

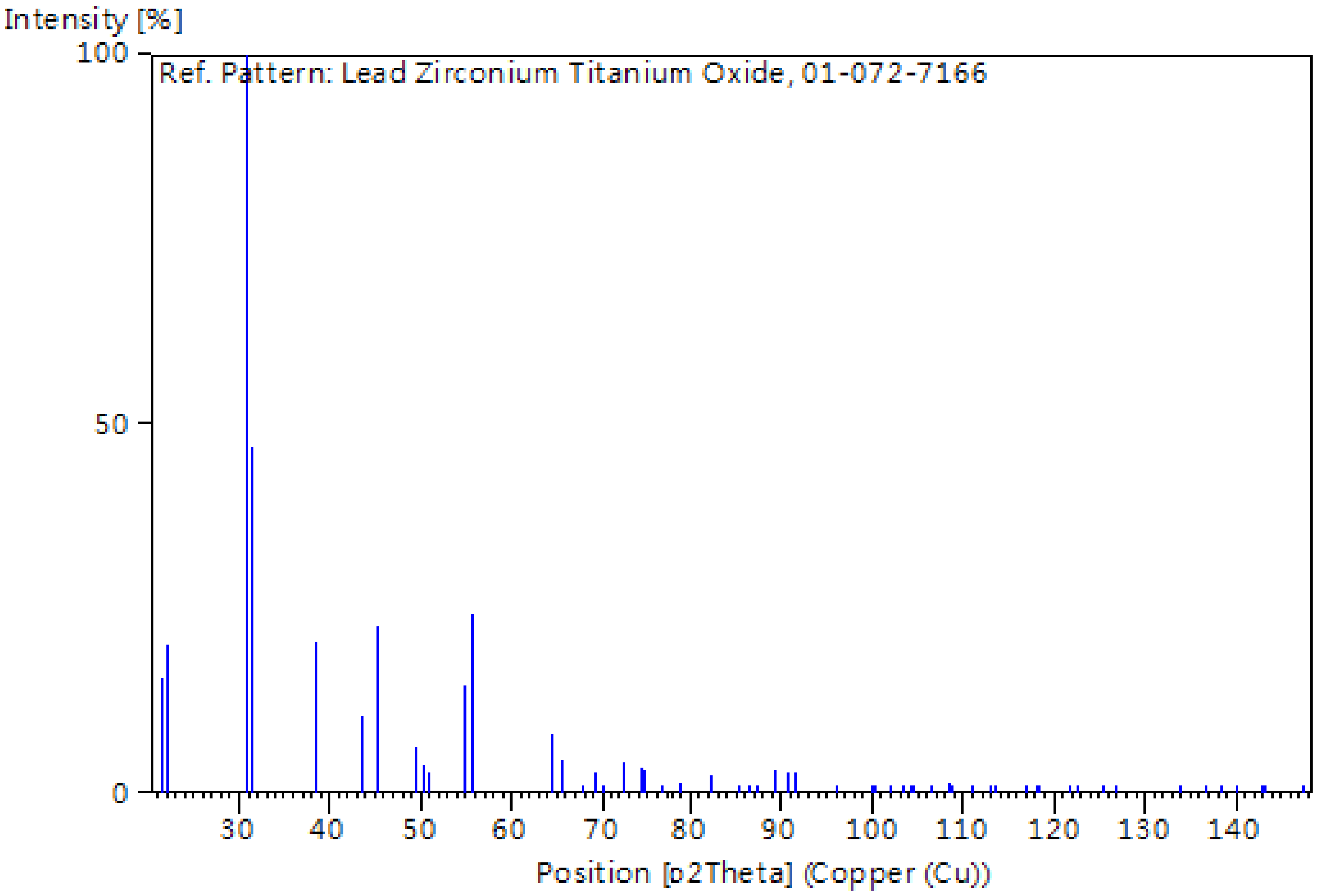

Supplement: XRD code dataset [file rsos171363supp11.pdf]
