# Supplementary material for: Hydrothermally synthesized PZT film grown in highly concentrated KOH solution with large electromechanical coupling coefficient for resonator
Source: R Soc Open Sci. 2017 Dec 20;4(12):171363. doi: 10.1098/rsos.171363 (PMC5750027; doi:10.1098/rsos.171363)

Name and formula

Reference code:	01-070-4259
Compound name:	Lead Zirconium Titanium Oxide
Empirical formula:	$O_3PbTi_{0.9}Zr_{0.1}$
Chemical formula:	$Pb (Zr_{0.1}Ti_{0.9}) O_3$

Crystallographic parameters

Crystal system:	Tetragonal
Space group:	P4mm
Space group number:	99
a (A):	3.9272
b (A):	3.9272
c (A):	4.1319
Alpha (°):	90.0000
Beta (°):	90.0000
Gamma (°):	90.0000
Volume of cell (10^6 pm^3):	63.73
Z:	1.00
RIR:	12.31

Subfiles and quality

Subfiles:	ICSD Pattern
	Inorganic
Quality:	Star (S)

Comments

ANX:	ABX3
ICSD collection code:	90694
Creation Date:	7/27/2010
Modification Date:	1/17/2013
ANX:	ABX3
Analysis:	O3 Pb1 Ti0.9 Zr0.1
Formula from original source:	Pb (Zr0.1 Ti0.9) O3
ICSD Collection Code:	90694
Wyckoff Sequence:	c b2 a(P4MM)
Unit Cell Data Source:	Powder Diffraction.

References

Primary reference: *Calculated from ICSD using POWD-12++*
Structure: Joseph, J., Vimala, T.M., Sivasubramanian, V., Murthy, V.R.K., *J. Mater. Sci.*, **35**, 1571, (2000)

Peak list

No.	h	k	l	d [A]	2Theta[deg]	I [%]
1	0	0	1	4.13190	21.489	18.9
2	1	0	0	3.92720	22.623	30.0
3	1	0	1	2.84660	31.400	100.0
4	1	1	0	2.77700	32.208	45.0
5	1	1	1	2.30480	39.050	30.5
6	0	0	2	2.06600	43.782	10.9
7	2	0	0	1.96360	46.194	24.0
8	1	0	2	1.82840	49.833	10.0
9	2	0	1	1.77350	51.486	7.2
10	2	1	0	1.75630	52.028	6.6
11	1	1	2	1.65760	55.383	18.0
12	2	1	1	1.61630	56.925	32.0
13	2	0	2	1.42330	65.532	10.9
14	2	2	0	1.38850	67.390	6.2
15	0	0	3	1.37730	68.012	1.0
16	2	1	2	1.33810	70.293	6.2
17	2	2	1	1.31620	71.641	2.5
18	3	0	0	1.30910	72.090	1.2
19	1	0	3	1.29970	72.694	7.4
20	3	0	1	1.24790	76.235	5.9
21	3	1	0	1.24190	76.670	5.7
22	1	1	3	1.23390	77.259	1.8
23	3	1	1	1.18930	80.736	4.8
24	2	2	2	1.15240	83.892	4.7
25	2	0	3	1.12760	86.178	1.8
26	3	0	2	1.10580	88.310	1.5
27	3	2	0	1.08920	90.018	1.2
28	2	1	3	1.08380	90.590	7.3
29	3	1	2	1.06440	92.721	6.6
30	3	2	1	1.05320	94.006	6.3
31	0	0	4	1.03300	96.437	0.6
32	1	0	4	0.99900	100.900	1.3
33	4	0	0	0.98180	103.364	1.6
34	2	2	3	0.97780	103.959	1.1
35	1	1	4	0.96820	105.424	2.6
36	3	2	2	0.96350	106.161	2.0
37	4	0	1	0.95520	107.497	0.8
38	4	1	0	0.95250	107.941	0.8
39	3	0	3	0.94890	108.541	2.5
40	4	1	1	0.92810	112.192	4.5
41	3	3	0	0.92560	112.654	1.2
42	3	1	3	0.92230	113.272	1.5
43	2	0	4	0.91420	114.830	1.8
44	3	3	1	0.90330	117.027	1.0
45	2	1	4	0.89040	119.792	2.1
46	4	0	2	0.88680	120.599	2.3
47	4	2	0	0.87810	122.621	2.6
48	4	1	2	0.86500	125.877	1.6
49	4	2	1	0.85900	127.466	1.4
50	3	2	3	0.85430	128.758	4.3
51	3	3	2	0.84470	131.544	2.1
52	2	2	4	0.82880	136.688	1.6

53	0	0	5	0.82640	137.534	0.3
54	3	0	4	0.81090	143.585	1.0
55	4	2	2	0.80820	144.768	4.6
56	1	0	5	0.80820	144.768	4.6
57	4	0	3	0.79950	148.934	0.9

Stick Pattern

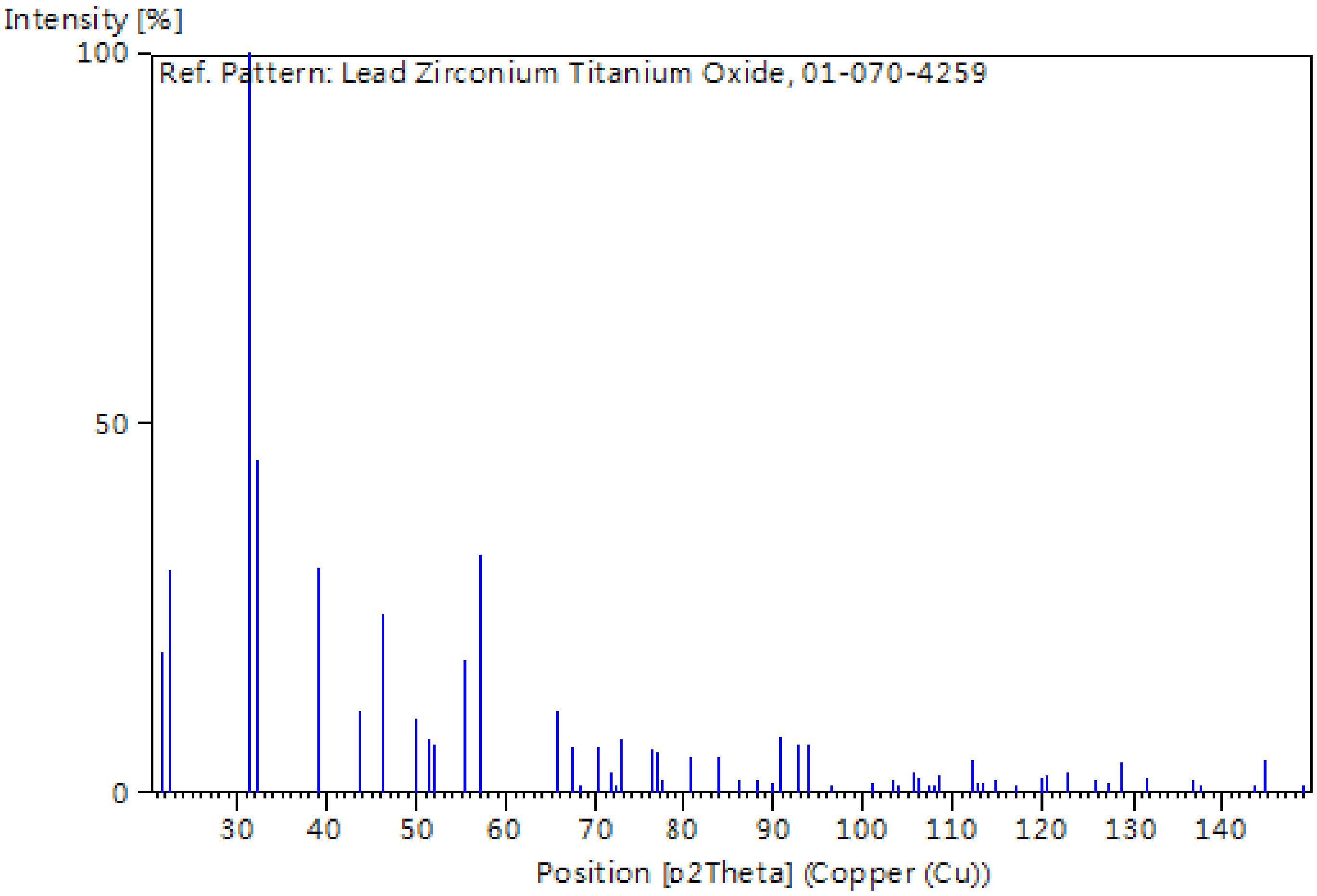

Supplement: XRD code dataset [file rsos171363supp12.pdf]
