# Supplementary material for: Hydrothermally synthesized PZT film grown in highly concentrated KOH solution with large electromechanical coupling coefficient for resonator
Source: R Soc Open Sci. 2017 Dec 20;4(12):171363. doi: 10.1098/rsos.171363 (PMC5750027; doi:10.1098/rsos.171363)

Name and formula

Reference code:	01-070-4260
Compound name:	Lead Zirconium Titanium Oxide
Empirical formula:	$O_3PbTi_{0.8}Zr_{0.2}$
Chemical formula:	$Pb (Zr_{0.2}Ti_{0.8}) O_3$

Crystallographic parameters

Crystal system:	Tetragonal
Space group:	P4mm
Space group number:	99
a (A):	3.9539
b (A):	3.9539
c (A):	4.1319
Alpha (°):	90.0000
Beta (°):	90.0000
Gamma (°):	90.0000
Volume of cell (10^6 pm^3):	64.60
Z:	1.00
RIR:	12.55

Status, subfiles and quality

Status:	Alternate Pattern
Subfiles:	ICSD Pattern
	Inorganic
Quality:	Star (S)

Comments

ANX:	ABX3
ICSD collection code:	90695
Creation Date:	7/27/2010
Modification Date:	1/17/2013
ANX:	ABX3
Analysis:	O3 Pb1 Ti0.8 Zr0.2
Formula from original source:	Pb (Zr0.2 Ti0.8) O3
ICSD Collection Code:	90695
Wyckoff Sequence:	c b2 a(P4MM)
Unit Cell Data Source:	Powder Diffraction.

References

Primary reference: *Calculated from ICSD using POWD-12++*
Structure: Joseph, J., Vimala, T.M., Sivasubramanian, V., Murthy, V.R.K., *J. Mater. Sci.*, **35**, 1571, (2000)

Peak list

No.	h	k	l	d [A]	2Theta[deg]	I [%]
1	0	0	1	4.13190	21.489	17.0
2	1	0	0	3.95390	22.468	27.3
3	1	0	1	2.85670	31.287	100.0
4	1	1	0	2.79580	31.986	45.3
5	1	1	1	2.31560	38.860	28.0
6	0	0	2	2.06600	43.782	10.9
7	2	0	0	1.97700	45.863	24.3
8	1	0	2	1.83110	49.755	9.0
9	2	0	1	1.78330	51.183	6.6
10	2	1	0	1.76820	51.652	5.9
11	1	1	2	1.66150	55.242	18.4
12	2	1	1	1.62560	56.570	32.9
13	2	0	2	1.42830	65.274	11.1
14	2	2	0	1.39790	66.877	6.3
15	0	0	3	1.37730	68.012	0.9
16	2	1	2	1.34340	69.975	5.6
17	2	2	1	1.32420	71.142	2.2
18	3	0	0	1.31800	71.528	1.1
19	1	0	3	1.30060	72.636	7.4
20	3	0	1	1.25560	75.685	6.1
21	3	1	0	1.25030	76.063	5.9
22	1	1	3	1.23550	77.140	1.6
23	3	1	1	1.19670	80.135	4.4
24	2	2	2	1.15780	83.413	4.7
25	2	0	3	1.13010	85.941	1.6
26	3	0	2	1.11110	87.780	1.3
27	3	2	0	1.09660	89.247	1.0
28	2	1	3	1.08660	90.292	7.4
29	3	1	2	1.06970	92.127	6.8
30	3	2	1	1.05990	93.232	6.5
31	0	0	4	1.03300	96.437	0.6
32	1	0	4	0.99940	100.845	1.2
33	4	0	0	0.98850	102.386	1.7
34	2	2	3	0.98110	103.467	1.0
35	1	1	4	0.96900	105.300	3.7
36	3	2	2	0.96900	105.300	3.7
37	4	0	1	0.96130	106.511	0.8
38	4	1	0	0.95900	106.880	0.7
39	3	0	3	0.95220	107.991	2.5
40	4	1	1	0.93410	111.105	4.6
41	3	3	0	0.93190	111.500	1.2
42	3	1	3	0.92580	112.617	1.3
43	2	0	4	0.91550	114.576	1.8
44	3	3	1	0.90910	115.843	0.9
45	4	0	2	0.89170	119.505	3.6
46	2	1	4	0.89170	119.505	3.6
47	4	2	0	0.88410	121.216	2.7
48	4	1	2	0.86980	124.652	1.4
49	4	2	1	0.86450	126.007	1.2
50	3	2	3	0.85790	127.764	4.3
51	3	3	2	0.84950	130.125	2.1

52	2	2	4	0.83080	135.998	1.6
53	0	0	5	0.82640	137.534	0.2
54	3	0	4	0.81280	142.780	4.9
55	4	2	2	0.81280	142.780	4.9
56	1	0	5	0.80890	144.457	2.0
57	4	0	3	0.80310	147.137	0.8

Stick Pattern

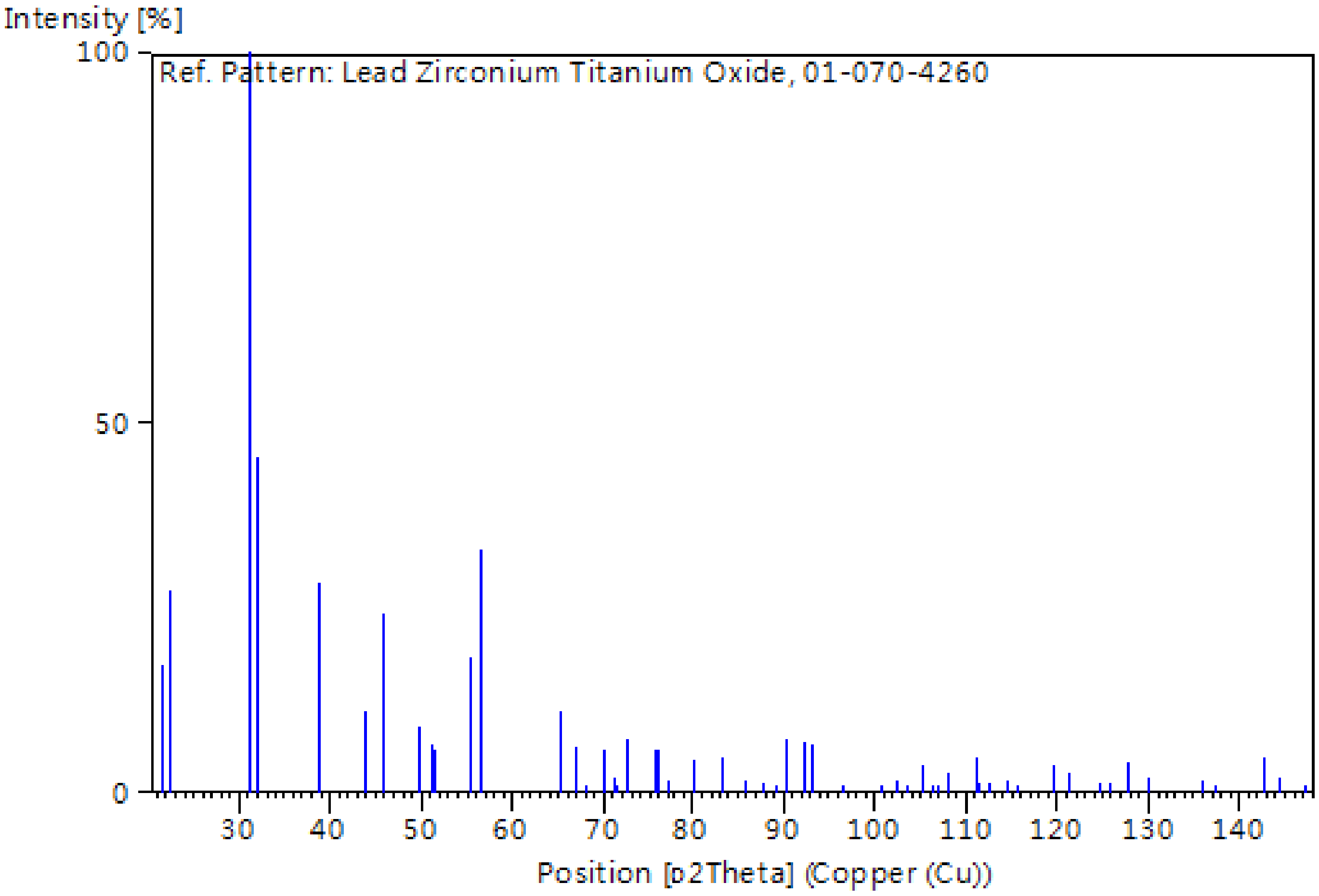

Supplement: XRD code dataset [file rsos171363supp13.pdf]
