# Supplementary material for: Hydrothermally synthesized PZT film grown in highly concentrated KOH solution with large electromechanical coupling coefficient for resonator
Source: R Soc Open Sci. 2017 Dec 20;4(12):171363. doi: 10.1098/rsos.171363 (PMC5750027; doi:10.1098/rsos.171363)

Name and formula

Reference code:	01-076-0326
Compound name:	Titanium Oxide
Common name:	Rutile, syn
Empirical formula:	O_2Ti
Chemical formula:	TiO_2

Crystallographic parameters

Crystal system:	Tetragonal
Space group:	P42/mnm
Space group number:	136
a (A):	4.6570
b (A):	4.6570
c (A):	3.0093
Alpha (°):	90.0000
Beta (°):	90.0000
Gamma (°):	90.0000
Volume of cell (10^6 pm^3):	65.26
Z:	2.00
RIR:	3.14

Status, subfiles and quality

Status:	Diffraction data collected at non ambient temperature
	Alternate Pattern
Subfiles:	Alloy, metal or intermetalic
	Common Phase
	Excipient
	Forensic
	ICSD Pattern
	Inorganic
	Mineral
	Pharmaceutical
Quality:	Indexed (I)

Comments

ANX:	AX2
ICSD collection code:	33846
Creation Date:	7/26/2010
Modification Date:	1/17/2013

ANX: AX2
Analysis: O2 Ti1
Formula from original source: Ti O2
ICSD Collection Code: 33846
Calculated Pattern Original Remarks: Stable from 800 to 1800 K (2nd ref., Tomaszewski), 300-800 K: Pbca. Temperature of Data Collection: 1883 K. Minor Warning: 7%<R factor<12% (for single crystal). Wyckoff Sequence: f a(P42/MNM). Unit Cell Data Source: Single Crystal.

References

Primary reference: *Calculated from ICSD using POWD-12++*, (2004)
Structure: Sugiyama, K., Takeuchi, Y., *Z. Kristallogr.*, **194**, 305, (1991)

Peak list

No.	h	k	l	d [A]	2Theta[deg]	I [%]
1	1	1	0	3.29300	27.056	100.0
2	1	0	1	2.52750	35.488	44.8
3	2	0	0	2.32850	38.636	6.2
4	1	1	1	2.22140	40.579	16.9
5	2	1	0	2.08270	43.414	6.1
6	2	1	1	1.71250	53.463	42.6
7	2	2	0	1.64650	55.788	10.5
8	0	0	2	1.50460	61.589	5.9
9	3	1	0	1.47270	63.075	4.6
10	2	2	1	1.44440	64.458	0.3
11	3	0	1	1.37960	67.884	10.8
12	1	1	2	1.36860	68.505	6.1
13	3	1	1	1.32280	71.229	0.5
14	3	2	0	1.29160	73.224	0.2
15	2	0	2	1.26380	75.108	1.2
16	2	1	2	1.21960	78.337	0.7
17	3	2	1	1.18690	80.933	1.5
18	4	0	0	1.16420	82.853	1.0
19	4	1	0	1.12950	85.998	0.4
20	2	2	2	1.11070	87.820	2.6
21	3	3	0	1.09770	89.134	1.1
22	4	1	1	1.05750	93.508	1.7
23	3	1	2	1.05250	94.088	1.7
24	4	2	0	1.04130	95.420	0.8
25	3	3	1	1.03120	96.661	0.1
26	4	2	1	0.98410	103.025	0.2
27	1	0	3	0.98060	103.541	0.7
28	3	2	2	0.98060	103.541	0.7
29	1	1	3	0.95960	106.783	0.2
30	4	3	0	0.93140	111.591	0.1
31	4	0	2	0.92080	113.556	0.6
32	5	1	0	0.91330	115.007	0.6
33	2	1	3	0.90370	116.944	1.9
34	4	1	2	0.90370	116.944	1.9
35	4	3	1	0.88980	119.925	1.1
36	3	3	2	0.88680	120.599	0.9
37	5	1	1	0.87390	123.636	0.1
38	5	2	0	0.86480	125.929	0.1
39	2	2	3	0.85630	128.203	0.8
40	4	2	2	0.85630	128.203	0.8
41	3	0	3	0.84250	132.214	1.0

42	5	2	1	0.83110	135.896	1.1
43	3	1	3	0.82900	136.618	0.1
44	4	4	0	0.82320	138.696	0.1
45	5	3	0	0.79870	149.350	0.1

Stick Pattern

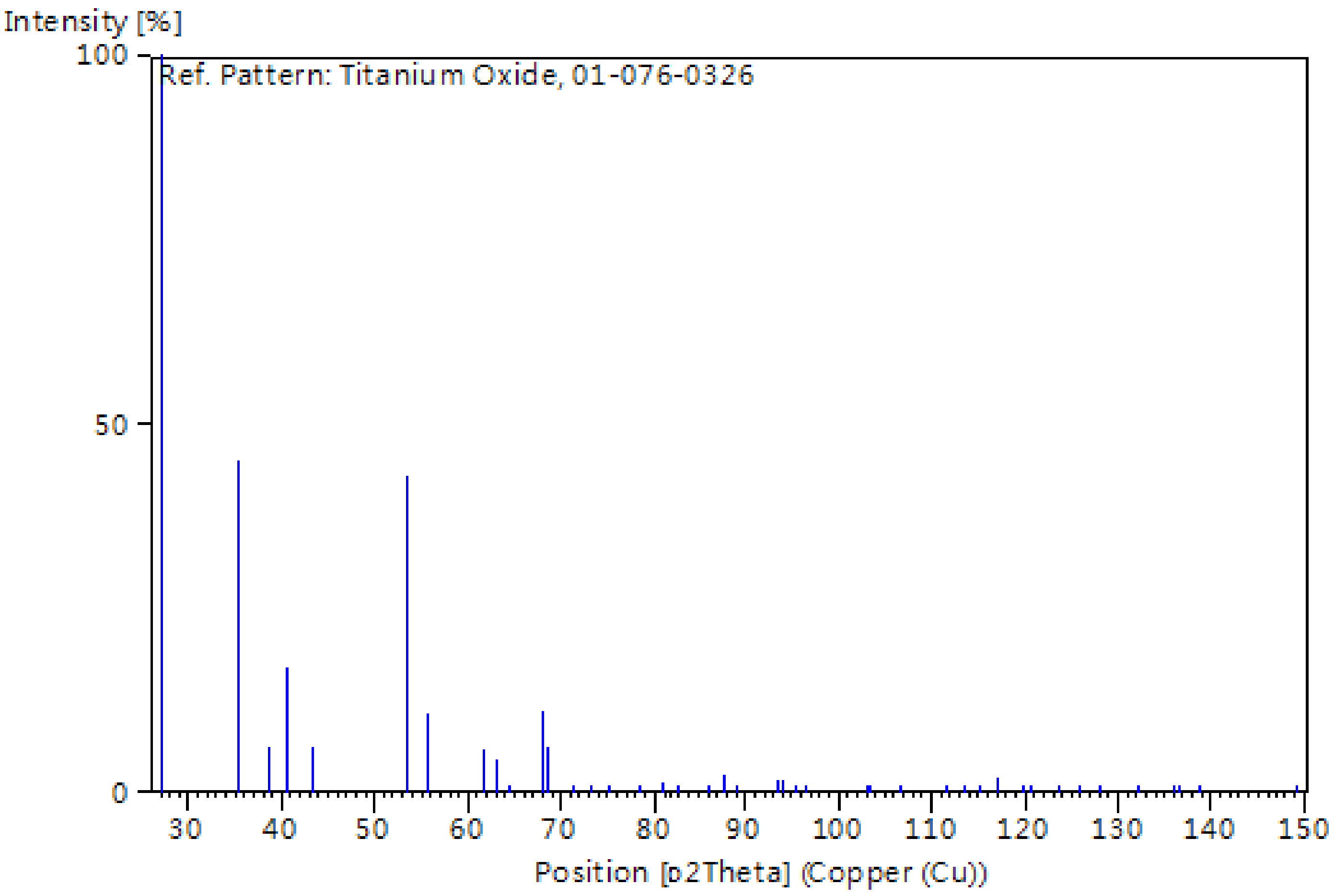

Supplement: XRD code dataset [file rsos171363supp14.pdf]
