# Supplementary material for: Hydrothermally synthesized PZT film grown in highly concentrated KOH solution with large electromechanical coupling coefficient for resonator
Source: R Soc Open Sci. 2017 Dec 20;4(12):171363. doi: 10.1098/rsos.171363 (PMC5750027; doi:10.1098/rsos.171363)
Supplement: XRD code dataset [file rsos171363supp15.pdf]

Name and formula

Reference code:	01-077-0856
Compound name:	Lead Zirconium Oxide
Common name:	lead zirconate
Empirical formula:	O_3PbZr
Chemical formula:	$PbZrO_3$

Crystallographic parameters

Crystal system:	Orthorhombic
Space group:	Cm2m
Space group number:	38
a (A):	5.8900
b (A):	5.8970
c (A):	4.1340
Alpha (°):	90.0000
Beta (°):	90.0000
Gamma (°):	90.0000
Volume of cell (10^6 pm^3):	143.59
Z:	2.00
RIR:	13.06

Subfiles and quality

Subfiles:	Ceramic
	ICSD Pattern
	Inorganic
Quality:	Star (S)

Comments

ANX:	ABX3
ICSD collection code:	39607
Creation Date:	7/27/2010
Modification Date:	1/17/2013
ANX:	ABX3
Analysis:	O3 Pb1 Zr1
Formula from original source:	Pb (Zr O3)
ICSD Collection Code:	39607
Calculated Pattern Original Remarks:	Stable above 503 K (2nd ref., Tomaszewski), below Pbma. The antiferromagnetic phase has a,b doubled, Pbma, cf. 59398
Wyckoff Sequence:	e b a2(CM2M)

Unit Cell Data Source: Single Crystal.

References

Primary reference: *Calculated from ICSD using POWD-12++*, (2004)
Structure: Shuvaeva, V.A., Antipin, M.Yu., Fesenko, O.E., Smotrakov, V.G., Struchkov, Yu.T., *Kristallografiya*, **37**, 1033, (1992)

Peak list

No.	h	k	l	d [A]	2Theta[deg]	I [%]
1	1	1	0	4.16730	21.304	13.0
2	0	0	1	4.13400	21.478	6.3
3	0	2	0	2.94850	30.289	43.2
4	2	0	0	2.94850	30.289	43.2
5	1	1	1	2.93490	30.432	100.0
6	0	2	1	2.40050	37.434	13.4
7	2	0	1	2.40050	37.434	13.4
8	2	2	0	2.08370	43.392	19.0
9	0	0	2	2.06700	43.760	13.1
10	3	1	0	1.86070	48.911	4.2
11	2	2	1	1.86070	48.911	4.2
12	1	1	2	1.85170	49.164	3.2
13	3	1	1	1.69970	53.898	21.6
14	1	3	1	1.69970	53.898	21.6
15	0	2	2	1.69250	54.146	15.8
16	2	0	2	1.69250	54.146	15.8
17	0	4	0	1.47420	63.003	3.1
18	4	0	0	1.47420	63.003	3.1
19	2	2	2	1.46740	63.329	9.9
20	0	4	1	1.38860	67.384	1.5
21	4	0	1	1.38860	67.384	1.5
22	3	1	2	1.38450	67.611	2.3
23	1	3	2	1.38450	67.611	2.3
24	0	0	3	1.37800	67.973	0.3
25	3	3	1	1.31680	71.603	5.1
26	4	2	0	1.31680	71.603	5.1
27	1	1	3	1.30830	72.141	6.5
28	4	2	1	1.25600	75.656	1.6
29	2	4	1	1.25600	75.656	1.6
30	2	0	3	1.24840	76.199	1.1
31	0	2	3	1.24840	76.199	1.1
32	0	4	2	1.20020	79.854	2.6
33	4	0	2	1.20020	79.854	2.6
34	1	5	0	1.15640	83.536	0.5
35	5	1	0	1.15520	83.643	0.3
36	3	3	2	1.15290	83.848	0.6
37	2	2	3	1.14940	84.161	0.7
38	1	5	1	1.11370	87.523	2.6
39	5	1	1	1.11260	87.632	1.8
40	2	4	2	1.11150	87.740	3.3
41	4	2	2	1.11150	87.740	3.3
42	1	3	3	1.10820	88.069	4.7
43	3	1	3	1.10820	88.069	4.7
44	4	4	0	1.04180	95.360	0.5
45	0	0	4	1.03350	96.375	0.8
46	5	3	0	1.01020	99.374	0.6
47	4	4	1	1.01020	99.374	0.6

48	1	5	2	1.00920	99.508	0.7
49	0	4	3	1.00670	99.845	0.4
50	4	0	3	1.00670	99.845	0.4
51	1	1	4	1.00310	100.335	0.4
52	3	5	1	0.98210	103.319	1.3
53	5	3	1	0.98210	103.319	1.3
54	3	3	3	0.97830	103.884	1.4
55	0	2	4	0.97530	104.335	2.2
56	2	0	4	0.97530	104.335	2.2
57	0	6	1	0.95620	107.334	0.3
58	6	0	1	0.95510	107.513	0.1
59	2	4	3	0.95260	107.924	0.6
60	4	2	3	0.95260	107.924	0.6
61	2	6	0	0.93230	111.428	0.6
62	6	2	0	0.93140	111.591	0.3
63	4	4	2	0.93030	111.790	0.8
64	2	2	4	0.92590	112.599	1.8
65	2	6	1	0.90940	115.783	0.5
66	3	5	2	0.90820	116.024	0.5
67	5	3	2	0.90820	116.024	0.5
68	3	1	4	0.90390	116.903	0.6
69	1	3	4	0.90390	116.903	0.6
70	0	6	2	0.88760	120.419	0.6
71	1	5	3	0.88580	120.827	1.4
72	5	1	3	0.88580	120.827	1.4
73	2	6	2	0.84980	130.038	1.0
74	6	2	2	0.84920	130.212	0.4
75	4	0	4	0.84630	131.065	1.1
76	0	4	4	0.84630	131.065	1.1
77	5	5	0	0.83390	134.956	0.3
78	1	7	0	0.83390	134.956	0.3
79	4	4	3	0.83100	135.930	0.3
80	3	3	4	0.82920	136.549	0.3
81	0	0	5	0.82680	137.391	0.1
82	5	5	1	0.81750	140.870	1.2
83	1	7	1	0.81750	140.870	1.2
84	5	3	3	0.81510	141.831	1.0
85	3	5	3	0.81510	141.831	1.0
86	4	2	4	0.81340	142.529	1.8
87	2	4	4	0.81340	142.529	1.8
88	1	1	5	0.81100	143.542	1.9
89	6	4	1	0.80190	147.723	0.3
90	4	6	1	0.80190	147.723	0.3
91	0	6	3	0.80020	148.576	0.3
92	6	0	3	0.79950	148.934	0.1

Stick Pattern

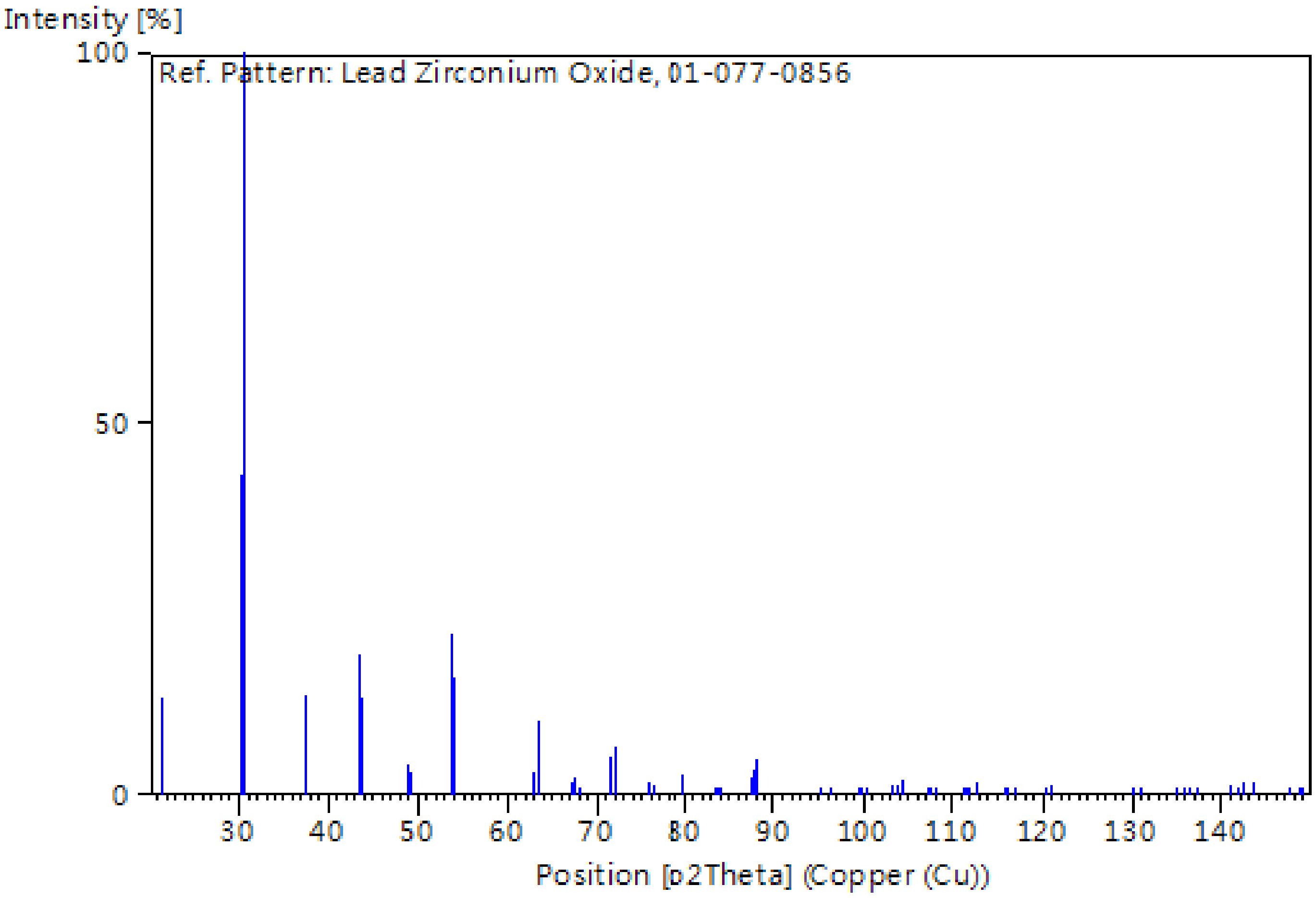
Intensity [%]
100
50
0
Ref. Pattern: Lead Zirconium Oxide, 01-077-0856
30
40
50
60
70
80
90
100
110
120
130
140
Position [°2Theta] (Copper (Cu))
