# Supplementary material for: Hydrothermally synthesized PZT film grown in highly concentrated KOH solution with large electromechanical coupling coefficient for resonator
Source: R Soc Open Sci. 2017 Dec 20;4(12):171363. doi: 10.1098/rsos.171363 (PMC5750027; doi:10.1098/rsos.171363)

Name and formula

Reference code: 01-074-2495

Mineral name: Macedonite, syn
Compound name: Lead Titanium Oxide
Common name: lead titanate

Empirical formula: O_3PbTi
Chemical formula: $Pb (TiO_3)$

Crystallographic parameters

Crystal system: Tetragonal
Space group: P4/mmm
Space group number: 123

a (A): 3.9000
b (A): 3.9000
c (A): 4.1500
Alpha (°): 90.0000
Beta (°): 90.0000
Gamma (°): 90.0000

Volume of cell (10^6 pm^3): 63.12
Z: 1.00

RIR: 11.20

Subfiles and quality

Subfiles: Ceramic
Common Phase
ICSD Pattern
Inorganic
Mineral
Quality: Indexed (I)

Comments

ANX: ABX3
ICSD collection code: 28624
Creation Date: 11/15/2010
Modification Date: 1/17/2013
ANX: ABX3
ICSD Collection Code: 28624
Calculated Pattern Original Remarks: Becomes cubic above 753 K. Given space group was P4mm, z`s should deviate from 0 and .5. Cell from 3rd ref

(Kabalkina & Vereshzhagin): 3.903, 4.154; at 17.8 kbar: 3.907, 4.051. Sample Source or Locality: synthetic at 1273 K. Temperature of Data Collection: 296 K. Minor Warning: No R factors reported/abstracted. Magnitude of e.s.d. on cell dimension is >1000 ppm. Unit Cell Data Source: Single Crystal.

References

Primary reference: *Calculated from ICSD using POWD-12++*, (2004)

Structure: Fedulov, S.A., Venevtsev, Y.N., Zhdanov, G.S., Smazhevskaya, E.G., Rez, I.S., *Kristallografiya*, **7**, 77, (1962)

Peak list

No.	h	k	l	d [A]	2Theta[deg]	I [%]
1	0	0	1	4.15000	21.394	20.4
2	1	0	0	3.90000	22.783	35.3
3	1	0	1	2.84200	31.453	100.0
4	1	1	0	2.75770	32.440	46.1
5	1	1	1	2.29680	39.191	39.4
6	0	0	2	2.07500	43.583	15.4
7	2	0	0	1.95000	46.535	24.2
8	1	0	2	1.83190	49.731	8.5
9	2	0	1	1.76490	51.756	7.4
10	2	1	0	1.74410	52.420	7.2
11	1	1	2	1.65810	55.365	16.7
12	2	1	1	1.60790	57.250	29.9
13	2	0	2	1.42100	65.651	13.4
14	0	0	3	1.38330	67.677	0.9
15	2	2	0	1.37890	67.923	5.8
16	2	1	2	1.33510	70.474	5.2
17	2	2	1	1.30850	72.128	2.5
18	1	0	3	1.30380	72.429	6.3
19	3	0	0	1.30000	72.675	1.3
20	3	0	1	1.24060	76.765	5.1
21	1	1	3	1.23650	77.066	3.6
22	3	1	0	1.23330	77.303	5.0
23	3	1	1	1.18220	81.322	5.6
24	2	2	2	1.14840	84.251	5.2
25	2	0	3	1.12830	86.111	1.3
26	3	0	2	1.10160	88.735	1.2
27	2	1	3	1.08380	90.590	5.8
28	3	2	0	1.08170	90.815	1.3
29	3	1	2	1.06020	93.198	5.3
30	3	2	1	1.04670	94.772	5.1
31	0	0	4	1.03750	95.882	0.9
32	1	0	4	1.00260	100.403	0.9
33	2	2	3	0.97660	104.139	0.9
34	4	0	0	0.97500	104.381	1.4
35	1	1	4	0.97110	104.976	2.0
36	3	2	2	0.95920	106.848	1.5
37	4	0	1	0.94920	108.490	0.8
38	3	0	3	0.94730	108.810	1.9
39	4	1	0	0.94590	109.048	0.8
40	4	1	1	0.92220	113.291	3.5
41	3	1	3	0.92060	113.594	2.4
42	3	3	0	0.91920	113.861	1.0
43	2	0	4	0.91590	114.498	2.4
44	3	3	1	0.89750	118.246	1.1
45	2	1	4	0.89170	119.505	1.3

46	4	0	2	0.88240	121.609	2.2
47	4	2	0	0.87210	124.079	2.1
48	4	1	2	0.86070	127.009	1.2
49	4	2	1	0.85340	129.011	1.3
50	3	2	3	0.85210	129.378	3.0
51	3	3	2	0.84050	132.833	1.5
52	0	0	5	0.83000	136.273	0.3
53	2	2	4	0.82900	136.618	2.0
54	1	0	5	0.81180	143.201	1.4
55	3	0	4	0.81090	143.585	0.7
56	4	2	2	0.80400	146.705	3.8

Stick Pattern

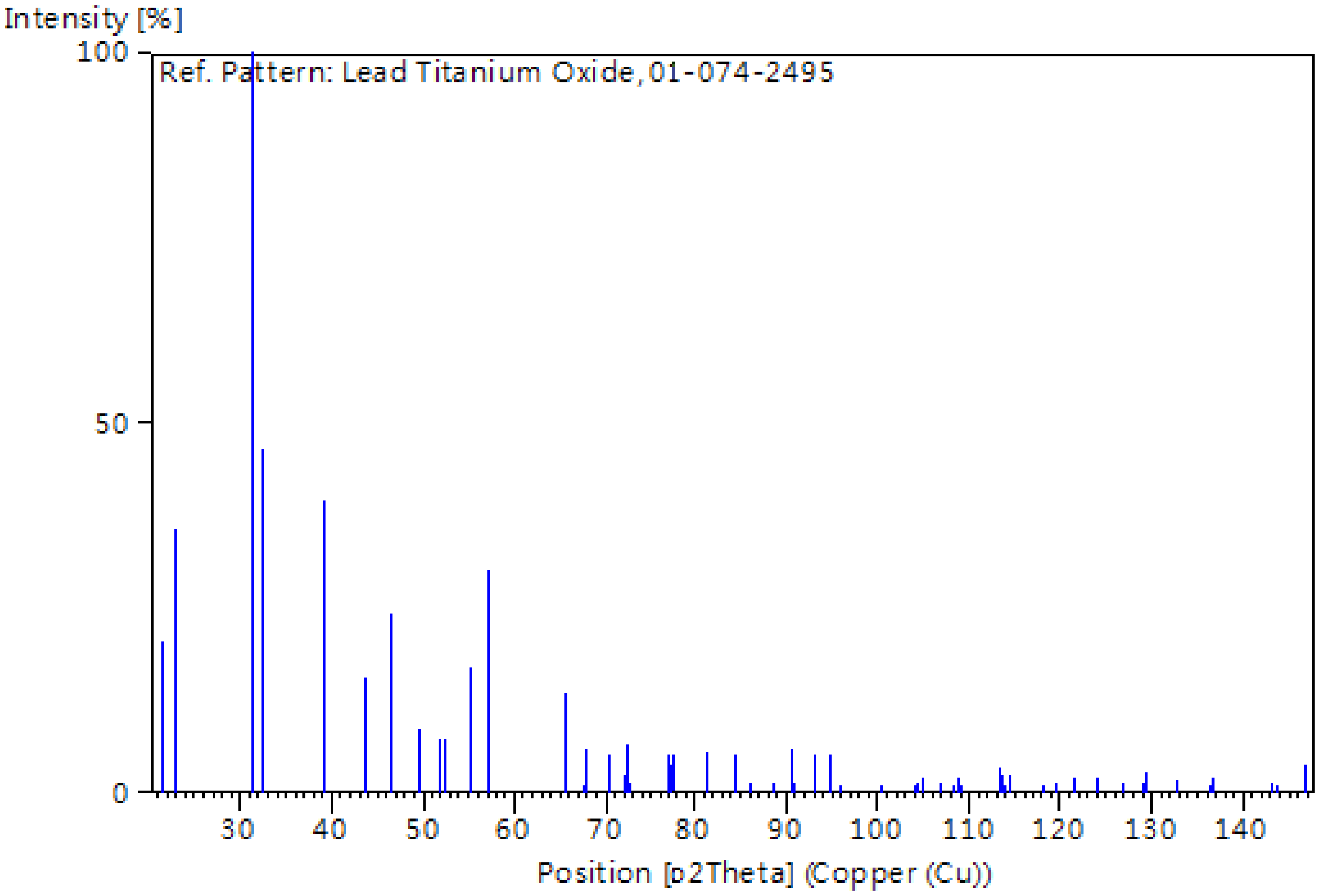

Supplement: XRD code dataset [file rsos171363supp16.pdf]
